# Supplementary material for: Rational design of isostructural 2D porphyrin-based covalent organic frameworks for tunable photocatalytic hydrogen evolution
Source: Nat Commun. 2021 Mar 1;12:1354. doi: 10.1038/s41467-021-21527-3 (PMC7921403; doi:10.1038/s41467-021-21527-3)
Supplement: Supplementary file 2 — Supplementary Information [file 41467_2021_21527_MOESM2_ESM.pdf]

Supplementary Information

Rational Design of Isostructural 2D Porphyrin-based Covalent Organic Frameworks for Tunable Photocatalytic Hydrogen Evolution - Chen *et al.*

Table of Contents

Section 1. Materials and Methods S3

Section 2. Synthetic Procedures S6

Section 3. Characterizations of MPor-DETH-COFs S8

Section 4. Structure Simulation S13

Section 5. Optical and electronic properties S23

Section 6. Photocatalysis S30

Section 7. DFT calculations S40

Section 8. NMR Spectrum S44

Section 9. References S45

Section 1. Materials and Methods

All the reagents and starting materials were purchased from Acros or Adamas, unless otherwise noted, and used without further purification. Dehydrated solvents were obtained after treating solvents with standard procedures. 2,5-diethoxyterephthalohydrazide (DETH)[1] and 5,10,15,20-tetrakis(4-benzaldehyde)porphyrin (*p*-Por-CHO)[2] were synthesized according to the literature.

^{1}H NMR and ^{13}C NMR spectra were recorded on a Bruker Fourier 400 M spectrometer. Solid-state ^{13}C NMR spectra was recorded at ambient temperature on a Bruker AVANCE III 400M spectrometer. High resolution MALDI-TOF data was collected on Bruker Solarix. Elemental analysis was collected on a Flash EA 1112. Fourier transform infrared (FT-IR) spectra were recorded on a NICOLET iN10 FTIR Spectrometer. Powder X-ray diffraction (PXRD) patterns were obtained on a Rigaku Smartlab or Rigaku MiniFlex 600 X-Ray diffractometer with Cu Kα line (λ = 1.5418 Å). Thermogravimetric analysis (TGA) from 20-800 °C was carried out on a TA Q500 in nitrogen/air atmosphere using a 10 °C min^{-1} ramp without equilibration delay. Field-emission scanning (FE-SEM) was performed on a Zeiss ΣIGMA operating at an accelerating voltage ranging from 0.1 to 20 kV. The sample was prepared by grinding and dispersing the material in ethanol and then dropped onto silicon wafers attached to a flat aluminum sample holder and then coated with gold. TEM was performed by JEM 2100F (operated at an accelerating voltage of 200 kV).

Molecular modeling was carried out using Materials Studio 8.0 suite of programs (Accelrys Inc.). The nitrogen isotherms were measured at 77 K using an Autosorb-iQ (Quantachrome) surface area size analyzer. Before measurement, the samples were degassed in vacuum at 120 °C for 24 h. Oil-free vacuum pumps and oil-free pressure regulators were used for measurements to prevent contamination of the samples during the degassing process and isotherm measurement. The Brunauer-Emmett-Teller (BET) method was utilized to calculate the specific surface areas. By using the quenched solid density functional theory (QSDFT) model, the pore size and volume were derived from

the sorption curve.

UV-vis spectra were recorded on a SHIMADZU UV-3600 UV-vis-NIR spectrophotometer. Cyclic voltammetry (CV) experiments were measured on a CHI 660E in a three-electrode electrochemical cell equipped with a salt bridge and a scan rate of 0.05 V s^{-1}. The experiments were conducted in anhydrous acetonitrile with tetrabutylammonium hexafluorophosphate (0.1 M) as supporting electrolyte. The auxiliary electrode was a platinum flakelet. The reference electrode was based on the Ag/AgCl couple. The working electrode was a glassy carbon electrode. Ferrocene/ferrocenium (Fc/Fc^+) redox potential was measured at the end of each experiment in order to calibrate the pseudo reference electrode as recommended by IUPAC. COF samples were dispersed in ethanol with a few droplets of 5 wt% Nafion and stirred for 3 h. The suspension was then dropped on the glassy carbon electrode and dried to form thin films for measurements. Valence band X-ray photoelectron spectra (VBXPS) were obtained on an ESCALAB 250Xi spectrophommeter with $Al\ K_\alpha$ radiation. The binding energy scale was calibrated using the C 1*s* peak at 284.60 eV.

Time-resolved emission decay was measured by time-correlated single photon counting (Edinburgh Instruments, FLS-920) with laser (405 nm) as the excitation light source. The photocurrent measurement experiments were conducted with a PGSTAT302N electrochemical workstation in a conventional three electrode cell, using a Pt plate as the counter electrode and an Ag/AgCl electrode as the reference electrode. The working electrode was prepared on indium-tin oxide (ITO) glass that was cleaned by sonication in ethanol for 30 min and dried at 353 K. The boundary of ITO glass was protected using Scotch tape. The sample (5 mg) was dispersed in ethanol (1 mL) by sonication to get a slurry. The slurry was spread onto pretreated ITO glass. After air-drying, the Scotch tape was unstuck, and the uncoated part of the electrode was isolated with epoxy resin.

X-ray Absorption spectroscopy measurement: X-ray Absorption spectroscopy (XAS) was acquired at beamline 1W1B at the Beijing Synchrotron Radiation Facility (BSRF) with Si (111) double-crystal monochromator. Under the condition of dedicated

synchrotron light, the ring energy is 2.5 GeV and the total beam current is 250 mA in top-up mode. Before XAS data collection, K-edge energy calibration was performed with corresponding metallic foil standards (Co, Ni, and Zn), and the data collection was carried out in transmission mode using ionization chamber. For MPor-DETH-COFs (M = Co, Ni, Zn), solid state samples were evenly milled and smeared onto a metal-free polyimide tape, and then the data was collected in fluorescence mode using a Lytle detector.

Extended X-ray absorption fine structure (EXAFS) spectra were transformed into *R*-space by Athena software. Firstly, the XAS spectra were obtained by subtracting the pre-edge background (−150 to −50 eV *vs*. absorption edge) from the overall absorption and then normalized with range of 150−700 eV. Subsequently, $\chi(k)$ data in the *k*-space were Fourier transformed to *R*-space using a hanning window (*k*-weight = 2, *k* is ranged from 3.0 to 12.0 $Å^{-1}$) to separate the EXAFS contributions from different coordination shells. EXAFS spectra were fitted by Artemis software and amplitude attenuation factor (amp) was calculated from corresponding metal foil. M−N and M−C single scattering paths were extracted from metal porphyrin model. The continuous Cauchy wavelet transform (CCWT) was carried out by using Larch 0.9.35 software (*k* weight = 2).

Section 2. Synthetic Procedures

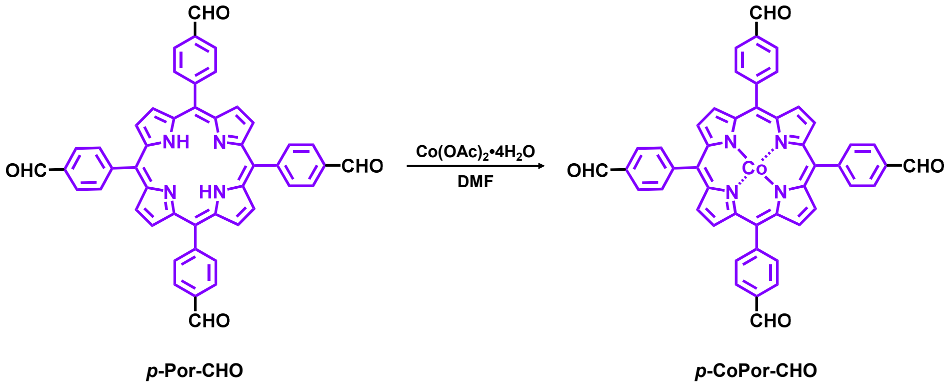

Supplementary Fig. 1 | Synthesis of *p*-CoPor-CHO.

Synthesis of *p*-CoPor-CHO: *p*-Por-CHO (400 mg, 0.55 mmol) and $Co(OAc)_2$•$4H_2O$ (200 mg, 0.80 mmol) were added to a flask containing DMF (50 mL). The reaction mixture was heated under nitrogen atmosphere at 120 ºC for 3 h. After cooling to room temperature, the mixture was poured into 500 mL of water. The residue was collected by filtration and then purified by column chromatography [SiO_2 : CH_2Cl_2 / EtOAc (20 : 1)] to yield product as a brown solid (348.5 mg, yield, 82%). HR-MS (MALDI-TOF) calculated for $C_{48}H_{28}CoN_4O_4$ m/z = 783.1443 $[M+H]^+$, found: m/z = 783.1441 $[M+H]^+$.

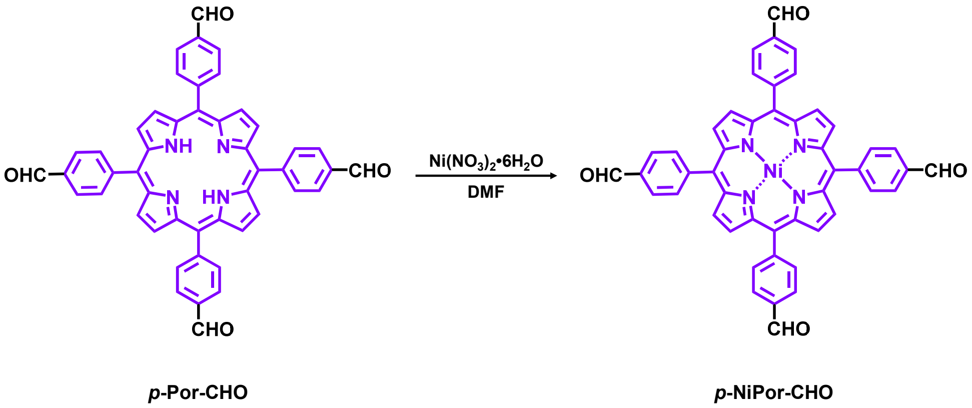

Supplementary Fig. 2 | Synthesis of *p*-NiPor-CHO.

Synthesis of *p*-NiPor-CHO: *p*-Por-CHO (400 mg, 0.55 mmol) and $Ni(NO_3)_2$•$6H_2O$ (232.63 mg, 0.80 mmol) were added to a flask containing DMF (100 mL). The reaction

mixture was heated under nitrogen atmosphere at 140 ºC for 1.5 h. After cooling to room temperature, the mixture was poured into 500 mL of water. The residue was collected by filtration and then purified by column chromatography [SiO_2 : CH_2Cl_2 / EtOAc (20 : 1)] to yield product as an amaranth solid (306 mg, yield, 71%). ^{1}H NMR (400 MHz, CD_2Cl_2, ppm): δ = 10.33 (s, 4H), 8.76 (s, 8H), 8.22 (s, 16H). HR-MS (MALDI-TOF) calculated for $C_{48}H_{28}NiN_4O_4$ m/z = 782.1464 $[M+H]^+$, found: m/z = 782.1462 $[M+H]^+$.

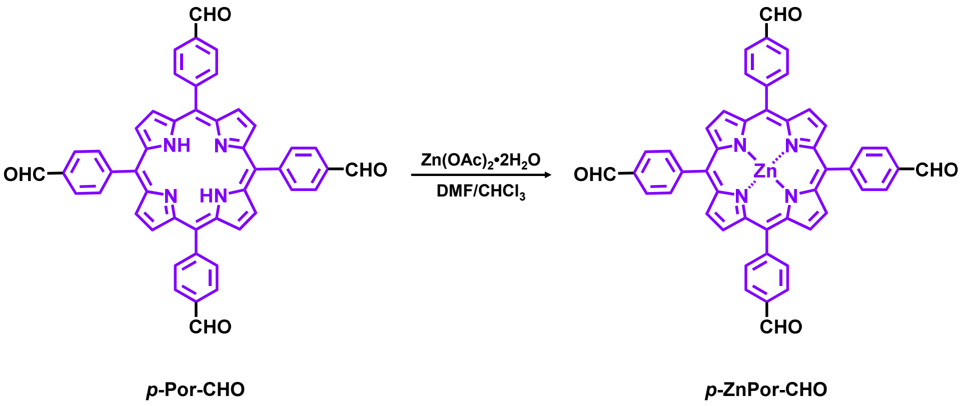

Supplementary Fig. 3 | Synthesis of *p*-ZnPor-CHO.

Synthesis of *p*-ZnPor-CHO: *p*-Por-CHO (400 mg, 0.55 mmol) and $Zn(OAc)_2•2H_2O$ (175.61 mg, 0.80 mmol) were added to a flask containing $CHCl_3$ (50 mL) and DMF (10 mL). The reaction mixture was heated under nitrogen atmosphere and refluxed for 3 h. After cooling to room temperature, the $CHCl_3$ solvent was removed under reduced pressure. Then the mixture was poured into 500 mL of water and the residue was collected by filtration and washed with 50 mL of methanol to yield product as a bright purple solid (425 mg, yield, 98%). ^{1}H NMR (400 MHz, CD_2Cl_2, ppm): δ = 10.38 (s, 4H), 8.96 (s, 8H), 8.41 (d, J = 8 Hz, 8H), 8.29 (d, J = 8 Hz, 8H). HR-MS (MALDI-TOF) calculated for $C_{48}H_{28}ZnN_4O_4$ m/z = 788.1402 $[M+H]^+$, found: m/z = 788.1401 $[M+H]^+$.

Section 3. Characterizations of MPor-DETH-COFs

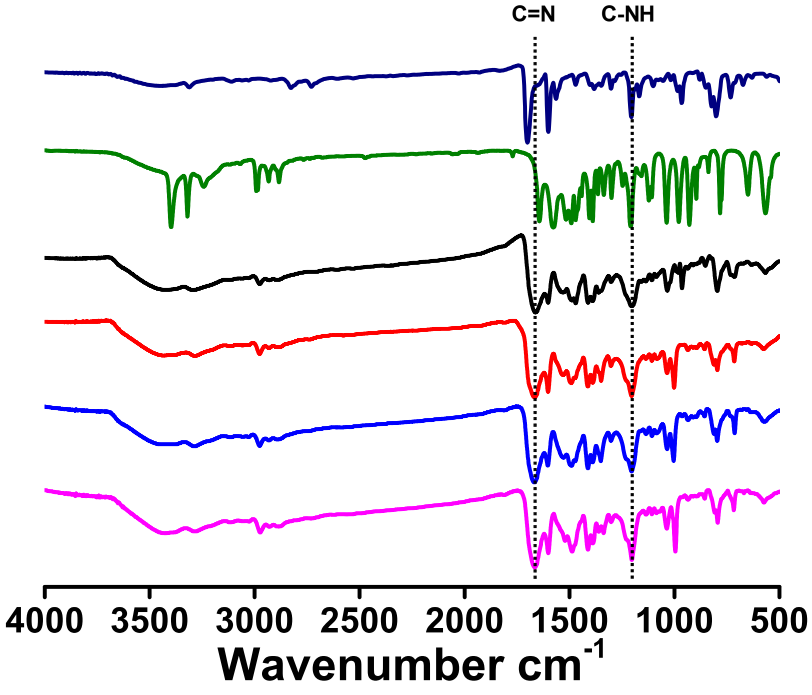

Supplementary Fig. 4 | FT-IR spectra of *p*-Por-CHO (navy curve), DETH (green curve), H_2Por-DETH-COF (black curve), CoPor-DETH-COF (red curve), NiPor-DETH-COF (blue curve) and ZnPor-DETH-COF (magenta curve).

B. ^{13}C solid-state NMR spectroscopy

High resolution solid-state NMR spectra were recorded at ambient pressure on a Bruker AVANCE III 400M spectrometer using a standard CP-TOSS pulse sequence (cross polarization with total suppression of sidebands) probe with 4 mm (outside diameter) zirconia rotors. Cross-polarization with TOSS was used to acquire ^{13}C data at 100.37 MHz. The ^{13}C ninety-degree pulse widths were 4 μs. The decoupling frequency corresponded to 72 kHz. The TOSS sample-spinning rate was 5 kHz. Recycle delays was 2s. The ^{13}C chemical shifts are given relative to glycine as 176.03 ppm.

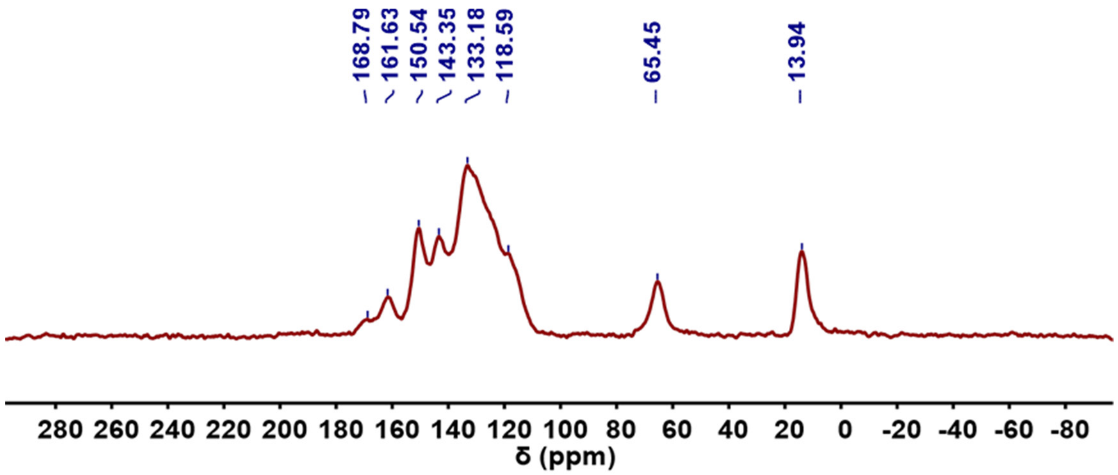

Supplementary Fig. 5 | Solid-state ^{13}C NMR spectra of H_2Por-DETH-COF.

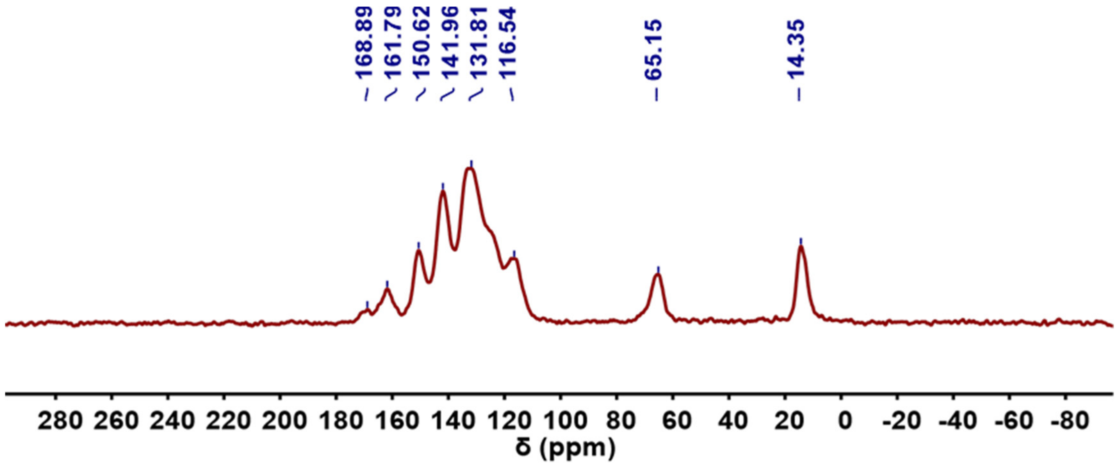

Supplementary Fig. 6 | Solid-state ^{13}C NMR spectra of NiPor-DETH-COF.

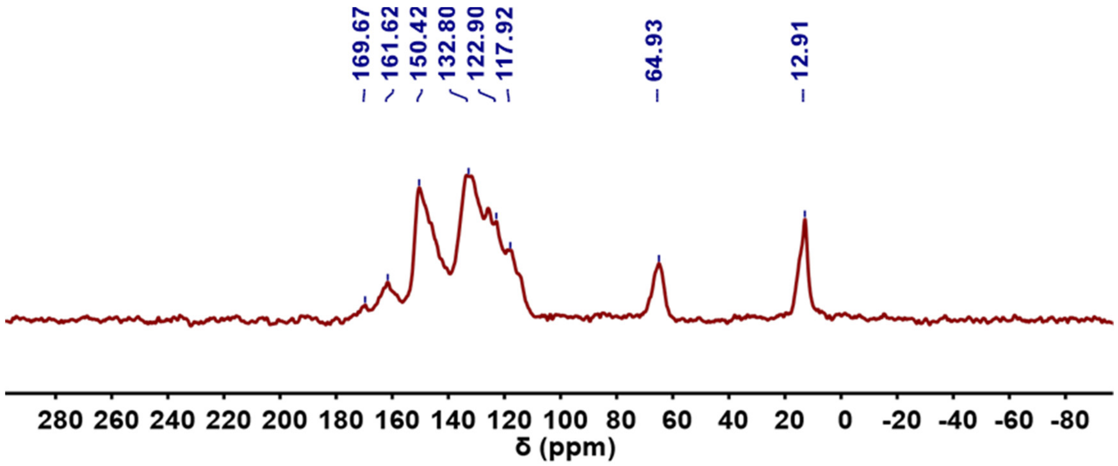

Supplementary Fig. 7 | Solid-state ^{13}C NMR spectra of ZnPor-DETH-COF.

C. Thermogravimetric analysis

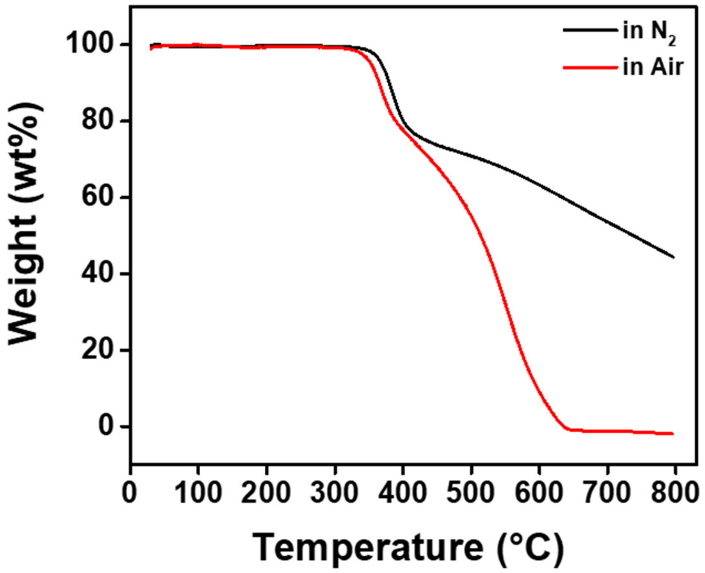

Supplementary Fig. 8 | TGA profile of H_2Por-DETH-COF in N_2 (black curve) and in air (red curve).

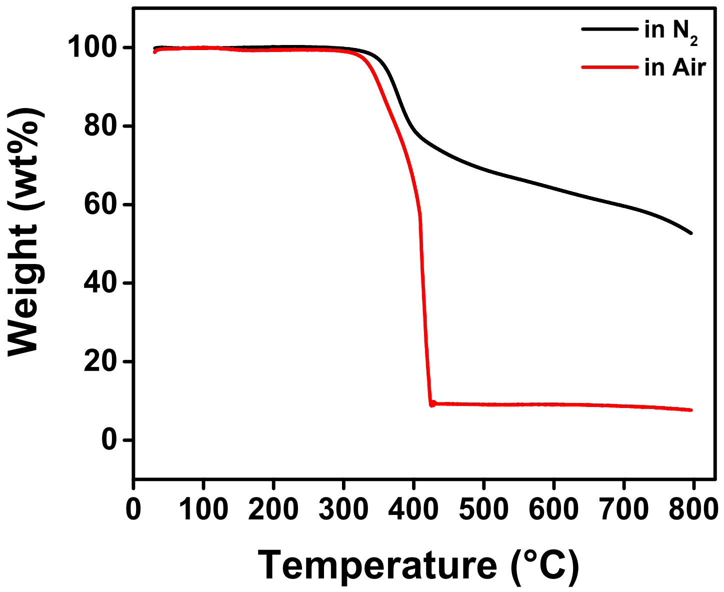

Supplementary Fig. 9 | TGA profile of CoPor-DETH-COF in N_2 (black curve) and in air (red curve).

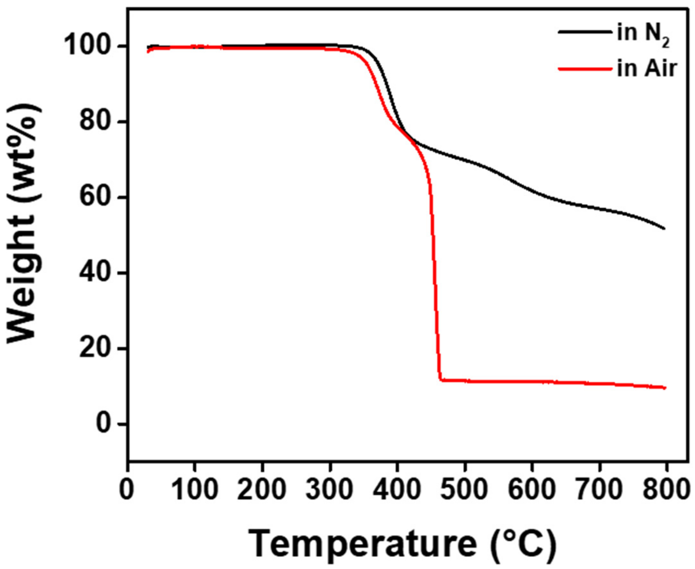

Supplementary Fig. 10 | TGA profile of NiPor-DETH-COF in N_2 (black curve) and in air (red curve).

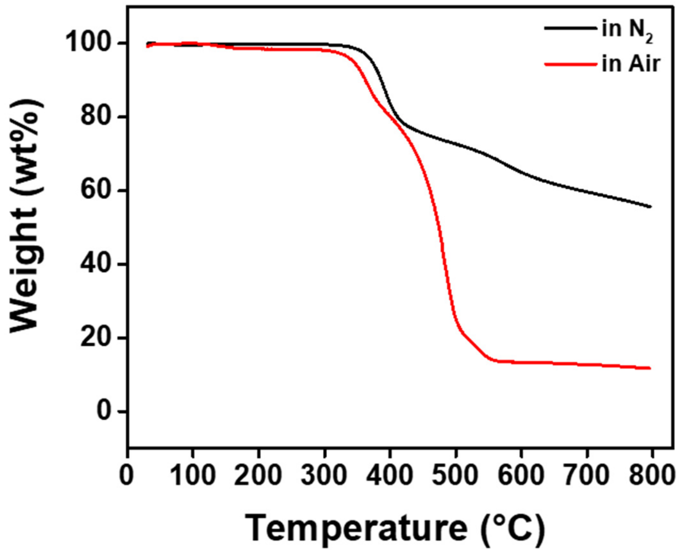

Supplementary Fig. 11 | TGA profile of ZnPor-DETH-COF in N_2 (black curve) and in air (red curve).

D. Scanning Electron Microscope Images

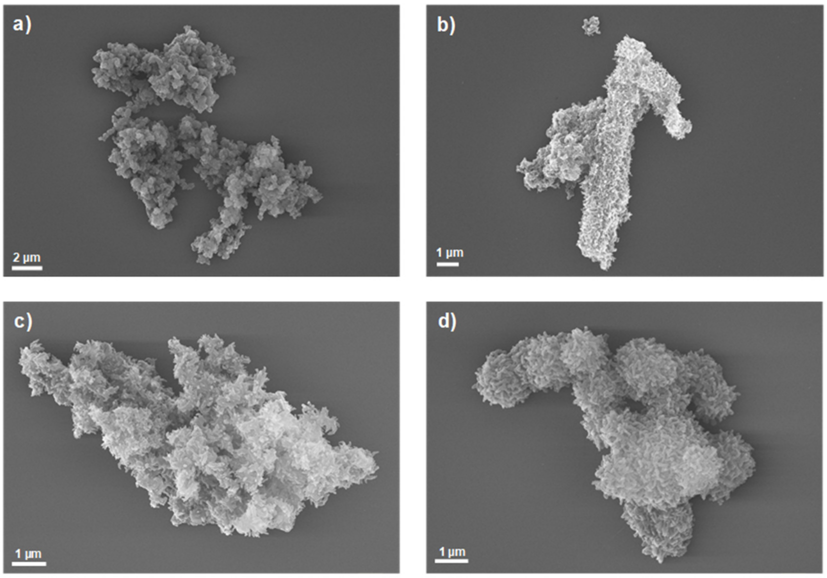

Supplementary Fig. 12 | SEM image of a) H_2Por-DETH-COF, b) CoPor-DETH-COF, c) NiPor-DETH-COF and d) ZnPor-DETH-COF.

E. Transmission Electron Microscope Images

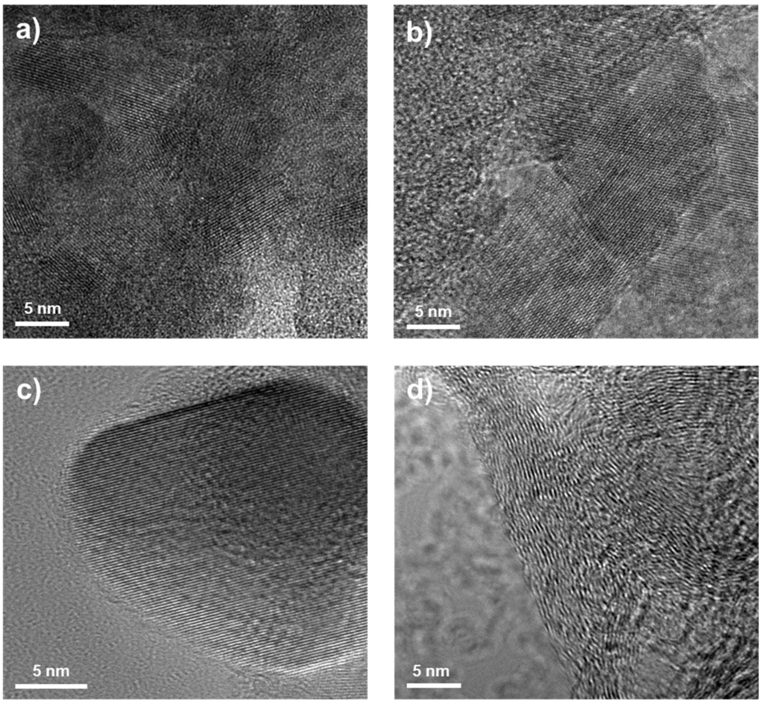

Supplementary Fig. 13 | TEM image of a) H_2Por-DETH-COF, b) CoPor-DETH-COF, c) NiPor-DETH-COF and d) ZnPor-DETH-COF.

Section 4. Structure Simulation

The unit cell structures of the eclipsed AA and staggered AB stacking modes were calculated using the density-functional tight-binding method in the Materials Studio software package. DFTB is an approximate density functional theory method based on the tight binding approach and utilizes an optimized minimal LCAO Slater-type all-valence basis set in combination with a two-center approximation for Hamiltonian matrix elements. The Coulombic interaction between partial atomic charges was determined using the self-consistent charge (SCC) formalism. Dispersion correction was employed in all calculations to describe van der Waals and π-stacking interactions.

The models of different COFs were generated using Materials Studio suite of programs. Vertex positions were obtained from the Reticular Chemistry Structure Resource.[3] Firstly, the eclipsed model was built and the symmetry of lattice was degraded to *P*1. Then the lattice model was fully optimized using the DFTB method. The staggered arrangement for the COFs was also examined by offsetting the alternating stacked units from the eclipsed model.

Pawley refinement was carried out using Reflex, a software package for crystal determination from XRD pattern. Unit cell dimension was set to the theoretical parameters. The Pawley refinement was performed to optimize the lattice parameters iteratively until the R_{wp} value converges and the overlay of the observed with refined profiles shows good agreement. The pseudo-Voigt profile function was used for whole profile fitting and Berrar–Baldinozzi function was used for asymmetry correction during the refinement processes. Line broadening from crystallite size and lattice strain were both considered.

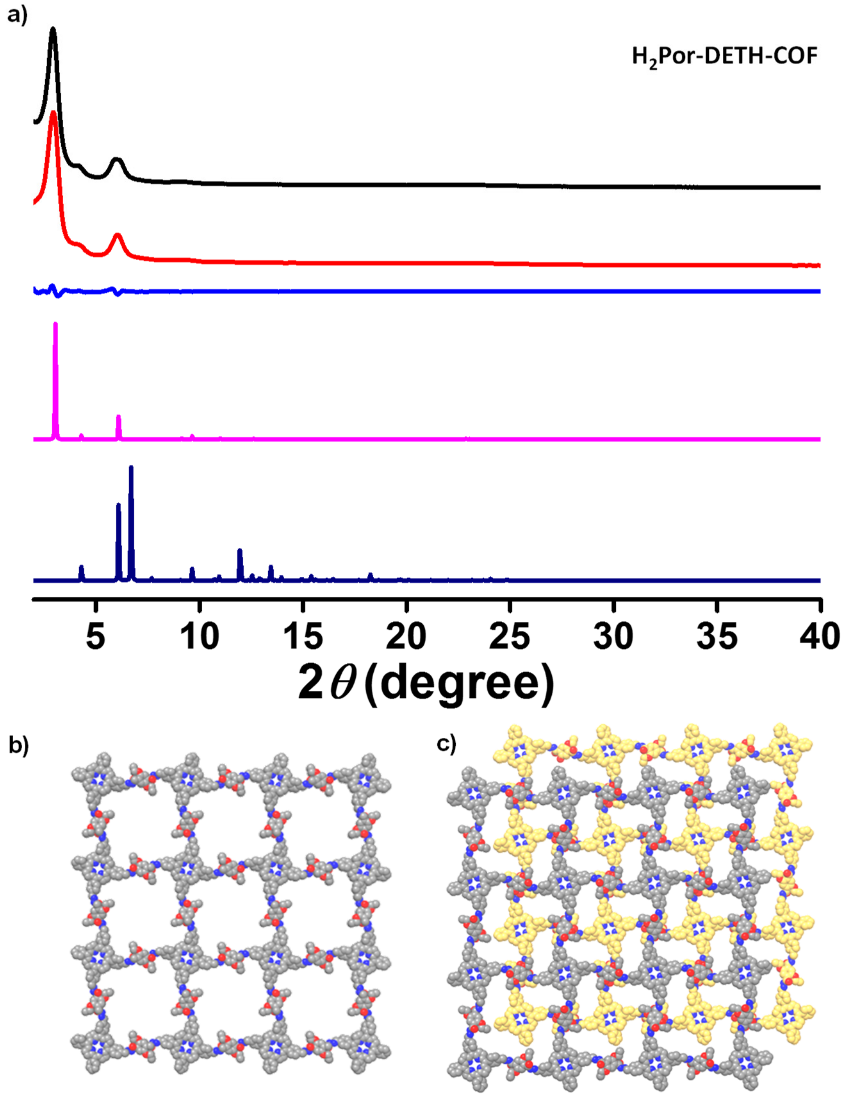

Supplementary Fig. 14 | a) PXRD pattern of the H_2Por-DETH-COF: experimental (black curve), Pawley refined (red curve), difference between experimental and calculated data (blue curve), calculated for AA-stacking (magenta curve), and calculated for AB stacking (navy curve). Space-filling models of the H_2Por-DETH-COF in b) AA and c) AB stacking modes. The Pawley refinement afforded a unit cell with parameters ($a = b = 40.9437$ Å, $c = 3.8889$ Å, $\alpha = \beta = \gamma = 90°$) that are very close to those of the suggested model ($a = b = 40.9420$ Å, $c = 3.8878$ Å, $\alpha = \beta = \gamma = 90°$). The final R_{wp} and R_p values converged to 3.16% and 2.35%, respectively.

Supplementary Table 1 | Atomistic coordinates for the Pawley-refined H_2Por-DETH-COF.

H_2Por-DETH-COF	Space group: *P*-4<br>$a = b = 40.9437$ Å, $c = 3.8889$ Å<br>$\alpha = \beta = \gamma = 90°$		
C	0.28283	0.26234	0.45119
C	0.21974	0.23972	0.50570
C	0.25869	0.28336	0.57359
C	0.24388	0.21871	0.38322
C	0.22642	0.27174	0.60553
C	0.27615	0.23033	0.35128
C	0.19879	0.29037	0.76330
C	0.30372	0.21166	0.19307
C	0.10805	0.36618	0.70293
C	0.39340	0.13505	0.24816
C	0.08921	0.39402	0.70470
C	0.41182	0.10693	0.24369
C	0.26478	0.37167	0.54116
C	0.23774	0.13041	0.41679
C	0.24620	0.34104	0.64020
C	0.25634	0.16101	0.31705
C	0.40272	0.16152	0.45298
C	0.09919	0.33943	0.49985
C	0.44018	0.10442	0.44202
C	0.06086	0.39596	0.50539
C	0.45016	0.13135	0.63629
C	0.05133	0.36874	0.31312
C	0.43156	0.15948	0.64327
C	0.07036	0.34090	0.30857
C	0.38048	0.18908	0.48807
C	0.12182	0.31218	0.46675
C	0.59450	-0.04306	0.58036
C	0.54290	0.09467	0.36145
C	0.60315	-0.01086	0.59608
C	0.51107	0.10303	0.34405
C	0.57488	0.00821	0.52319
C	0.49240	0.07370	0.42089
C	0.56056	-0.04482	0.50007
C	0.54401	0.06000	0.44470
C	0.45830	0.07319	0.45239

C	0.42693	-0.04233	0.49256
H	0.30756	0.27193	0.42208
H	0.19501	0.23013	0.53482
H	0.13050	0.36492	0.85391
H	0.37095	0.13677	0.09770
H	0.09635	0.41484	0.86282
H	0.40431	0.08640	0.08315
H	0.27608	0.36900	0.28879
H	0.22644	0.13313	0.66913
H	0.21846	0.12523	0.23102
H	0.28405	0.37683	0.72709
H	0.24833	0.39258	0.53319
H	0.25417	0.10948	0.42515
H	0.23488	0.34394	0.89761
H	0.26766	0.15805	0.05972
H	0.27592	0.16651	0.50460
H	0.22662	0.33556	0.45256
H	0.47201	0.12959	0.79625
H	0.02954	0.37003	0.15176
H	0.43902	0.18000	0.80464
H	0.06327	0.32017	0.14833
H	0.33313	0.22151	0.61142
H	0.16950	0.28029	0.34477
H	0.61051	-0.06375	0.62613
H	0.56364	0.11063	0.31418
H	0.62714	-0.00128	0.65946
H	0.50121	0.12685	0.27684
H	0.47416	0.00658	0.42235
H	0.38250	0.20501	0.71825
H	0.12005	0.29614	0.23716
O	0.26934	0.31440	0.64723
O	0.23322	0.18766	0.30932
O	0.20107	0.30588	1.03796
O	0.30136	0.19618	-0.08175
N	0.33397	0.21581	0.35081
N	0.16858	0.28604	0.60521
N	0.35674	0.19193	0.26805
N	0.14557	0.30970	0.68718
N	0.54956	-0.01327	0.47669
N	0.51300	0.04773	0.46948

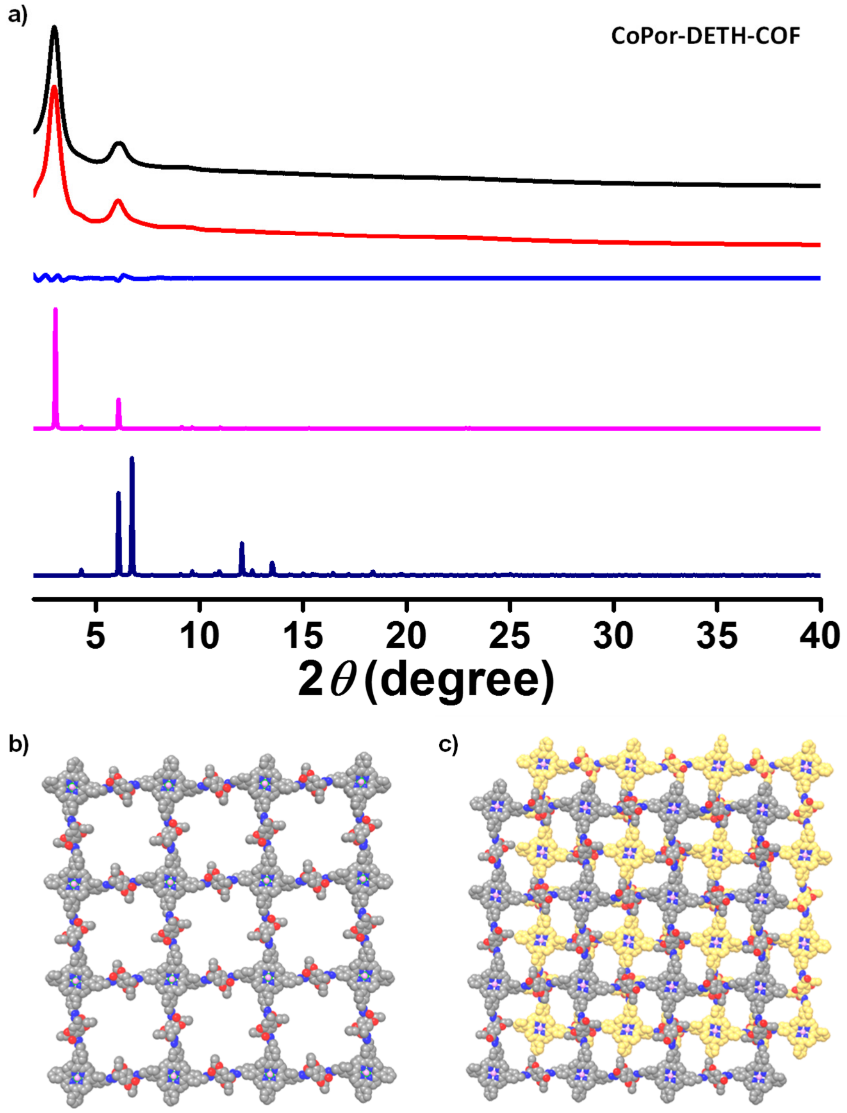

Supplementary Fig. 15 | a) PXRD pattern of the CoPor-DETH-COF: experimental (black curve), Pawley refined (red curve), difference between experimental and calculated data (blue curve), calculated for AA-stacking (magenta curve), and calculated for AB stacking (navy curve). Space-filling models of the CoPor-DETH-COF in b) AA and c) AB stacking modes. The Pawley refinement afforded a unit cell with parameters ($a = b = 40.9417$ Å, $c = 3.8917$ Å, $\alpha = \beta = \gamma = 90°$) that are very close to those of the suggested model ($a = b = 40.9420$ Å, $c = 3.8878$ Å, $\alpha = \beta = \gamma = 90°$). The final R_{wp} and R_p values converged to 1.59% and 1.11%, respectively.

Supplementary Table 2 | Atomistic coordinates for the Pawley-refined CoPor-DETH-COF.

CoPor-DETH-COF	Space group: *P*-4/*n*<br>$a = b = 40.9417$ Å, $c = 3.8917$ Å<br>$\alpha = \beta = \gamma = 90°$		
C	0.77826	0.25407	0.30899
C	0.76508	0.28170	0.47564
C	0.73629	0.27730	0.67657
C	0.71366	0.30387	0.81378
C	0.62219	0.37843	0.63716
C	0.60142	0.40525	0.63391
C	0.79717	0.36539	0.54639
C	0.77182	0.33907	0.61348
C	0.88538	0.14936	0.56325
C	0.92725	0.09564	0.56751
C	0.93509	0.12355	0.76299
C	0.91401	0.15028	0.76433
C	0.86216	0.17636	0.56250
C	1.09929	-0.02940	0.66060
C	1.10421	0.00346	0.60931
C	1.07294	0.01735	0.51295
C	1.06516	-0.03581	0.58626
C	0.94927	0.06717	0.56013
H	0.80058	0.25724	0.15350
H	0.64458	0.37840	0.79434
H	0.60667	0.42684	0.79327
H	0.80002	0.36986	0.26807
H	0.67882	0.14181	0.34909
H	0.78974	0.38845	0.67049
H	0.77021	0.33336	0.89398
H	0.75279	0.15331	0.48167
H	0.95732	0.12367	0.92176
H	0.91950	0.17194	0.92195
H	0.81656	0.21359	0.58460
H	1.11755	-0.04728	0.74549
H	1.12726	0.01680	0.64020
H	0.86396	0.19615	0.76023
O	0.78224	0.31000	0.42619
O	0.71812	0.32238	1.05319
N	0.81618	0.19918	0.36144
N	0.83871	0.17517	0.33774
N	1.04995	-0.00691	0.51073
Co	1.50000	0.50000	0.50000

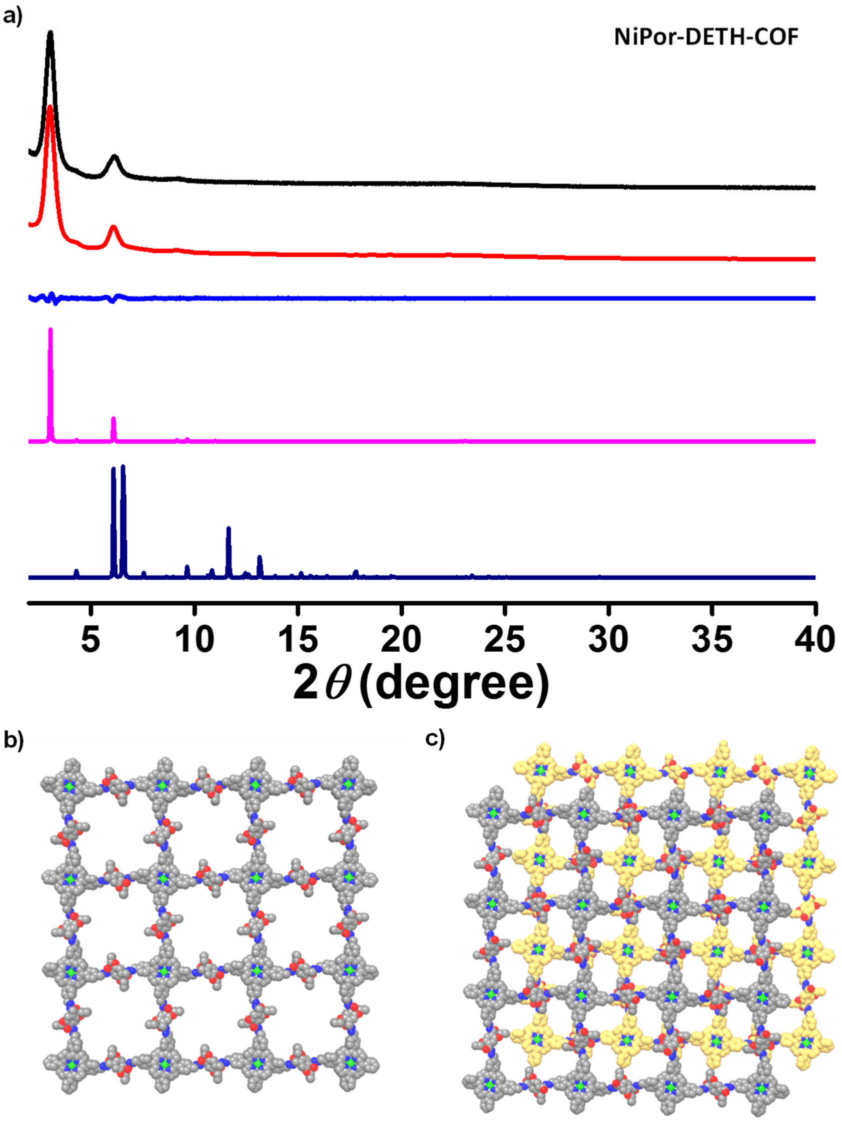

Supplementary Fig. 16 | a) PXRD pattern of the NiPor-DETH-COF: experimental (black curve), Pawley refined (red curve), difference between experimental and calculated data (blue curve), calculated for AA-stacking (magenta curve), and calculated for AB stacking (navy curve). Space-filling models of the NiPor-DETH-COF in b) AA and c) AB stacking modes. The Pawley refinement afforded a unit cell with parameters ($a = b = 40.9421$ Å, $c = 3.8889$ Å, $\alpha = \beta = \gamma = 90°$) that are very close to those of the suggested model ($a = b = 40.9420$ Å, $c = 3.8878$ Å, $\alpha = \beta = \gamma = 90°$). The final R_{wp} and R_p values converged to 3.93% and 2.96%, respectively.

Supplementary Table 3 | Atomistic coordinates for the Pawley-refined NiPor-DETH-COF.

NiPor-DETH-COF	Space group: *P*-4/*n*<br>$a = b = 40.9421$ Å, $c = 3.8889$ Å<br>$\alpha = \beta = \gamma = 90°$		
C	0.78193	0.26034	0.46005
C	0.75904	0.28189	0.60386
C	0.72638	0.27131	0.64910
C	0.69989	0.29073	0.81432
C	0.60699	0.36547	0.77230
C	0.58801	0.39354	0.76997
C	0.76781	0.37011	0.58844
C	0.74882	0.34007	0.68781
C	0.90170	0.16153	0.43260
C	0.94028	0.10456	0.43500
C	0.94972	0.13208	0.62794
C	0.93059	0.16019	0.62791
C	0.87876	0.18853	0.46724
C	1.09389	-0.04389	0.59366
C	1.10253	-0.01178	0.63129
C	1.07371	0.00740	0.57191
C	1.05951	-0.04476	0.51917
C	0.95840	0.07347	0.45593
H	0.80697	0.26927	0.42112
H	0.62942	0.36425	0.92672
H	0.59505	0.41459	0.92843
H	0.77781	0.36792	0.32765
H	0.71170	0.12581	0.23468
H	0.75207	0.39178	0.59618
H	0.73824	0.34281	0.94955
H	0.77134	0.16484	0.49556
H	0.97137	0.13080	0.79433
H	0.93748	0.18112	0.78947
H	0.83127	0.22234	0.58875
H	1.10997	-0.06508	0.61666
H	1.12666	-0.00215	0.69615
H	0.88150	0.20559	0.68926
O	0.77143	0.31241	0.68576
O	0.70293	0.30623	1.08565
N	0.83029	0.21199	0.34486
N	0.85359	0.18983	0.26483
N	1.04703	-0.01315	0.52047
Ni	1.50000	0.50000	0.50000

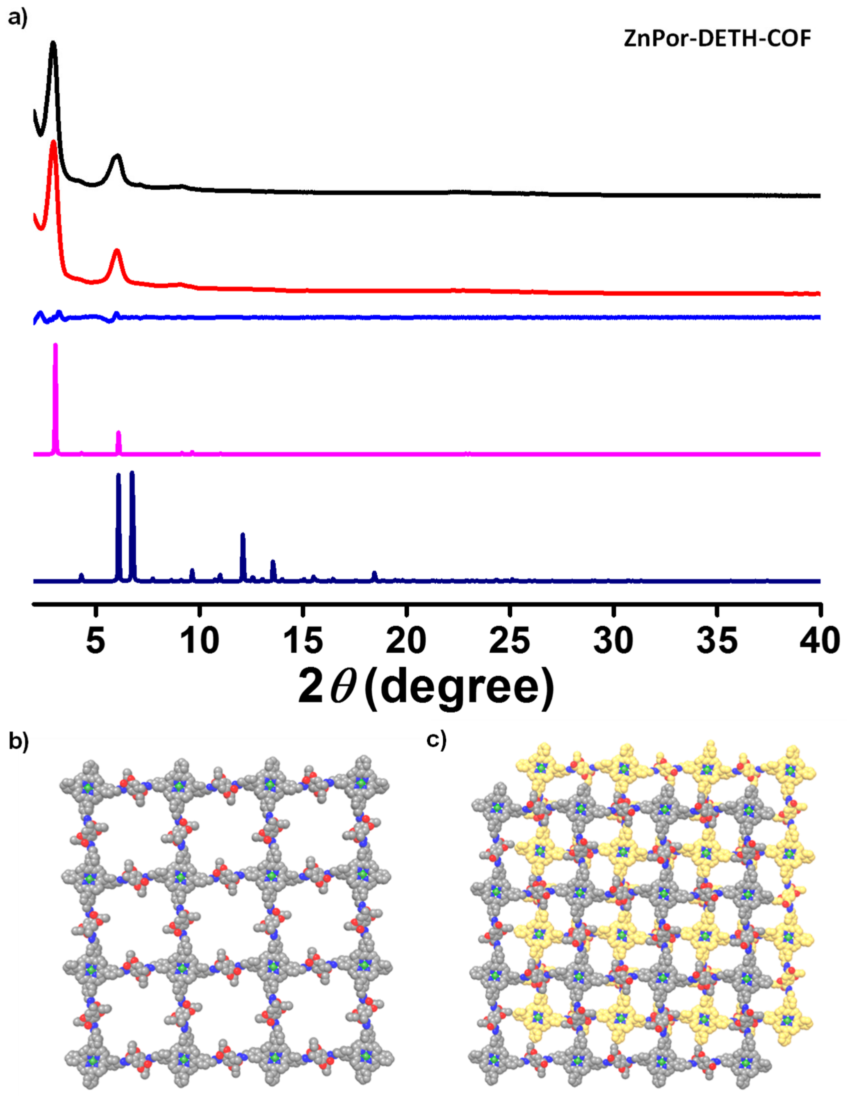

Supplementary Fig. 17 | a) PXRD pattern of the ZnPor-DETH-COF: experimental (black curve), Pawley refined (red curve), difference between experimental and calculated data (blue curve), calculated for AA-stacking (magenta curve), and calculated for AB stacking (navy curve). Space-filling models of the ZnPor-DETH-COF in b) AA and c) AB stacking modes. The Pawley refinement afforded a unit cell with parameters ($a = b = 40.9424$ Å, $c = 3.8894$ Å, $\alpha = \beta = \gamma = 90°$) that are very close to those of the suggested model ($a = b = 40.9420$ Å, $c = 3.8878$ Å, $\alpha = \beta = \gamma = 90°$). The final R_{wp} and R_p values converged to 4.34% and 3.19%, respectively.

**Supplementary Table 4 | **Atomistic coordinates for the Pawley-refined ZnPor-DETH-COF

ZnPor-DETH-COF	Space group: *P*-4/*n*<br>$a = b = 40.9424$ Å, $c = 3.8894$ Å<br>$\alpha = \beta = \gamma = 90°$		
C	0.78197	0.26090	0.47723
C	0.75783	0.28228	0.59984
C	0.72517	0.27115	0.62751
C	0.69793	0.29035	0.78094
C	0.60563	0.36401	0.73856
C	0.58697	0.39228	0.74200
C	0.76519	0.37008	0.53812
C	0.74597	0.34048	0.64655
C	0.90354	0.16270	0.46845
C	0.94149	0.10530	0.45989
C	0.95145	0.13254	0.65441
C	0.93262	0.16087	0.65998
C	0.88075	0.18982	0.50640
C	1.09374	-0.04489	0.60679
C	1.10287	-0.01281	0.62850
C	1.07406	0.00654	0.56427
C	1.05914	-0.04544	0.53373
C	0.95928	0.07399	0.47407
H	0.80701	0.27025	0.45107
H	0.62821	0.36237	0.89015
H	0.59436	0.41304	0.90289
H	0.77755	0.36595	0.29151
H	0.71611	0.12379	0.27000
H	0.74899	0.39124	0.50950
H	0.73373	0.34478	0.89769
H	0.77291	0.16603	0.54620
H	0.97332	0.13085	0.81732
H	0.93996	0.18155	0.82293
H	0.83273	0.22363	0.62458
H	1.10960	-0.06614	0.63990
H	1.12725	-0.00341	0.68651
H	0.88360	0.20682	0.72883
O	0.76895	0.31328	0.68149
O	0.70033	0.30641	1.04943
N	0.83204	0.21311	0.38142
N	0.85558	0.19111	0.30414
N	1.04765	-0.01389	0.52318
Zn	1.50000	0.50000	0.50000

Section 5. Optical and electronic properties

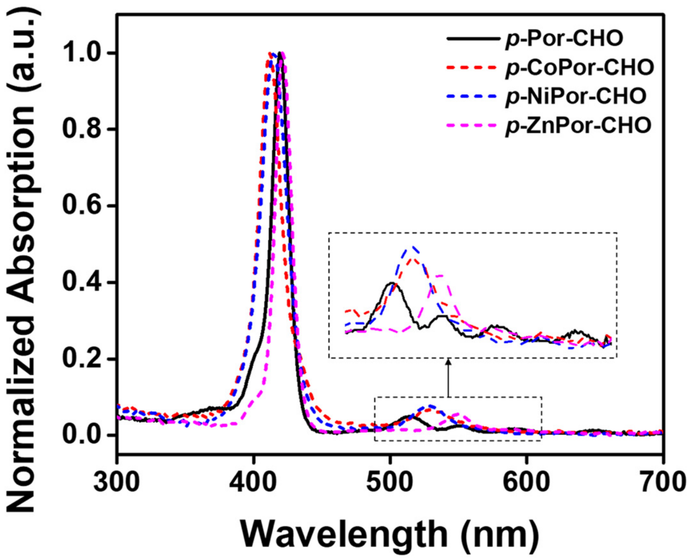

Supplementary Fig. 18 | UV-visible spectra of *p*-Por-CHO (black curve), *p*-CoPor-CHO (red curve), *p*-NiPor-CHO (blue curve) and *p*-ZnPor-CHO (magenta curve) at the concentration of 1.6×10^{-6} mol L^{-1} in DCM.

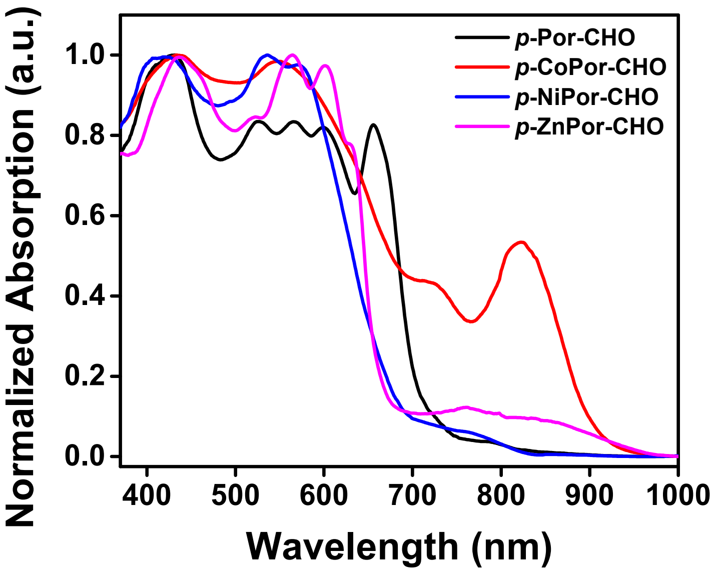

Supplementary Fig. 19 | UV-vis diffuse reflection absorption spectra of *p*-Por-CHO (black curve), *p*-CoPor-CHO (red curve), *p*-NiPor-CHO (blue curve) and *p*-ZnPor-CHO (magenta curve).

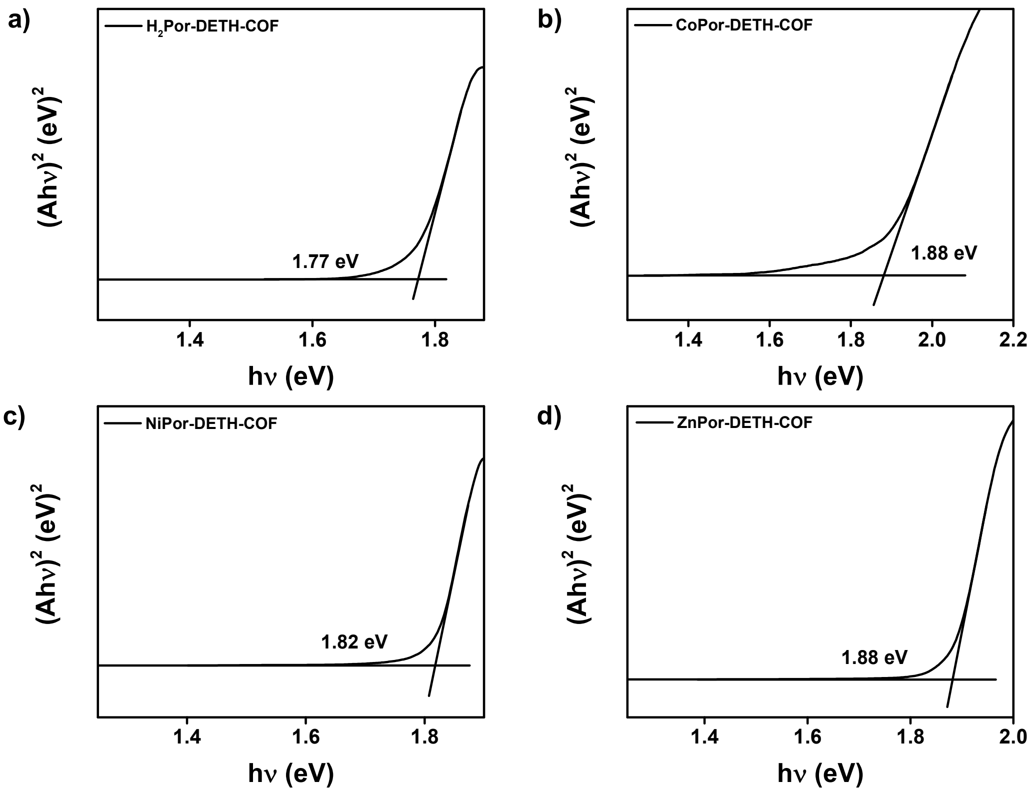

Supplementary Fig. 20 | Tauc plot for absorption spectra obtained with Kubelka-Munk function of a) H_2Por-DETH-COF, b) CoPor-DETH-COF, c) NiPor-DETH-COF, d) ZnPor-DETH-COF and the linear fit for direct band gaps.

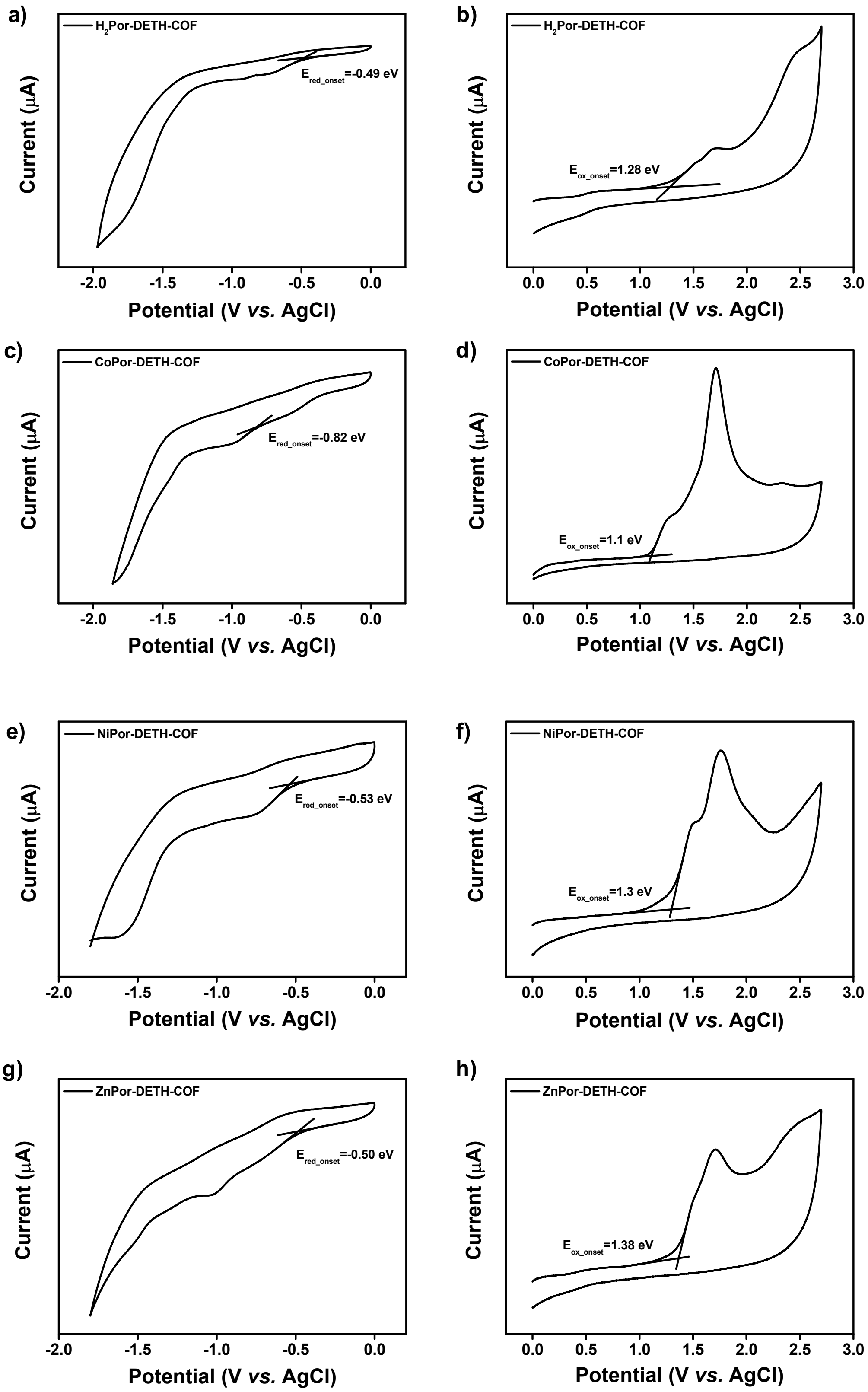

Supplementary Fig. 21 | Cyclic voltammetry measurements of H_2Por-DETH-COF (a and b), CoPor-DETH-COF (c and d), NiPor-DETH-COF (e and f) and ZnPor-DETH-COF (g and h).

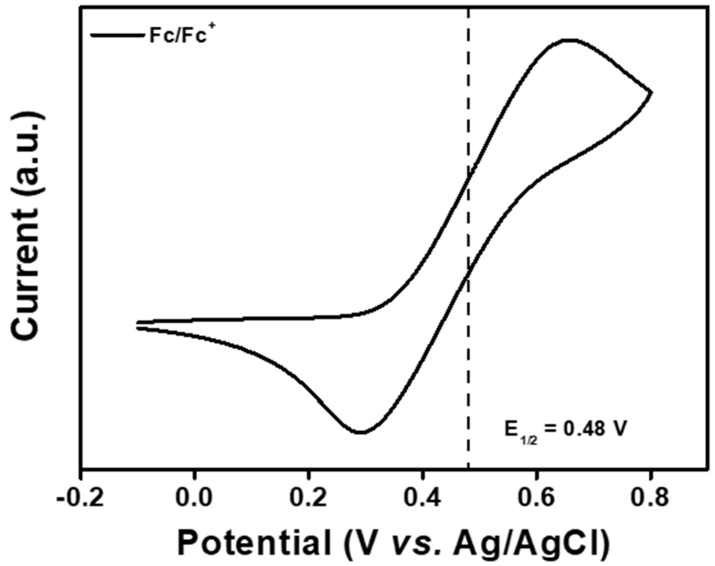

Supplementary Fig. 22 | Cyclic voltammetry measurements of ferrocene/ferrocenium couple to calibrate the pseudo reference electrode. The HOMO and LUMO levels of H_2Por-DETH-COF, CoPor-DETH-COF, NiPor-DETH-COF and ZnPor-DETH-COF. Ferrocene/ferrocenium (Fc/Fc^+) redox potential was measured at the end of each experiment to calibrate the reference electrode. The HOMO and LUMO levels are determined as follows: $E_{HOMO} = -[E_{ox} - E\ (Fc/Fc^+) + 4.8]$ eV and $E_{LUMO} = -[E_{red} - E\ (Fc/Fc^+) + 4.8]$ eV.

Supplementary Table 5 | Electronic and optical band structures of H_2Por-DETH-COF, CoPor-DETH-COF, NiPor-DETH-COF and ZnPor-DETH-COF.

	**Reduction Potential (V)**	**Oxidation Potential (V)**	**LUMO (eV)**	**HOMO (eV)**	**$Eg_{electronic}$ (eV)**	**$Eg_{optical}$ (eV)**
**H_2Por-DETH-COF**	-0.49	1.28	-3.83	-5.60	1.77	1.77
**CoPor-DETH-COF**	-0.82	1.10	-3.50	-5.42	1.92	1.88
**NiPor-DETH-COF**	-0.53	1.30	-3.79	-5.62	1.83	1.82
**ZnPor-DETH-COF**	-0.50	1.38	-3.82	-5.7	1.88	1.88

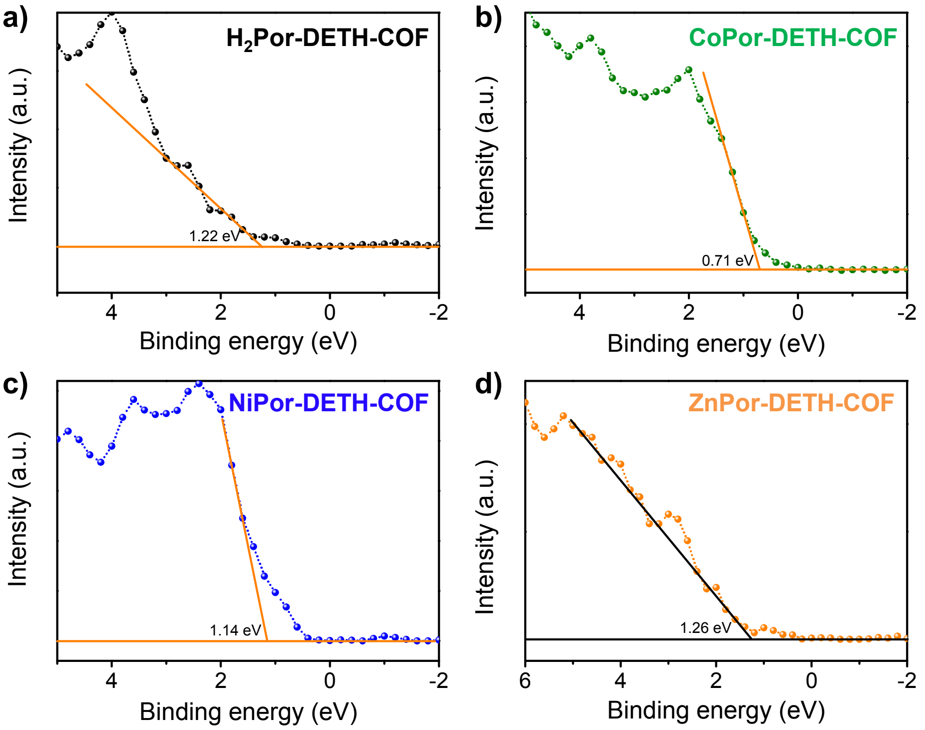

Supplementary Fig. 23 | Valence band XPS spectra of a) H_2Por-DETH-COF, b) CoPor-DETH-COF, c) NiPor-DETH-COF, d) ZnPor-DETH-COF. The binding energy scale was calibrated using the *C 1s* peak at 284.60 eV.

Supplementary Table 6 | Comparison of the results of the determination of HOMO by CV and valence band spectra of XPS.

Samples	HOMO *vs* vacuum (CV)	HOMO *vs* NHE (CV)	XPS (valence band) *vs* NHE
H_2Por-DETH-COF	–5.60	1.10	1.22
CoPor-DETH-COF	–5.42	0.92	0.71
NiPor-DETH-COF	–5.62	1.12	1.14
ZnPor-DETH-COF	–5.70	1.20	1.26

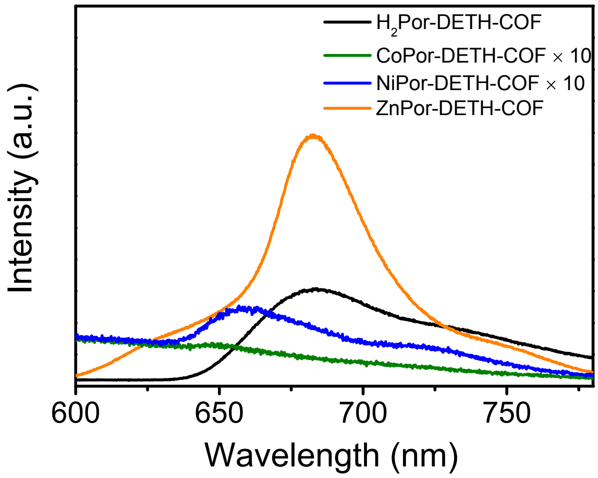

Supplementary Fig. 24 | PL spectra of all four MPor-DETH-COFs (Excitation: 405 nm).

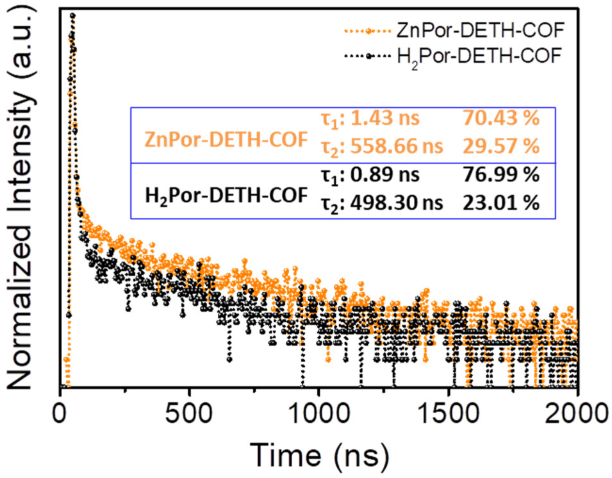

Supplementary Fig. 25 | Emission decay of H_2Por-DETH-COF and ZnPor-DETH-COF (Excitation: 405 nm; signal position: 682 nm; room temperature under the condition of oil pump vacuum).

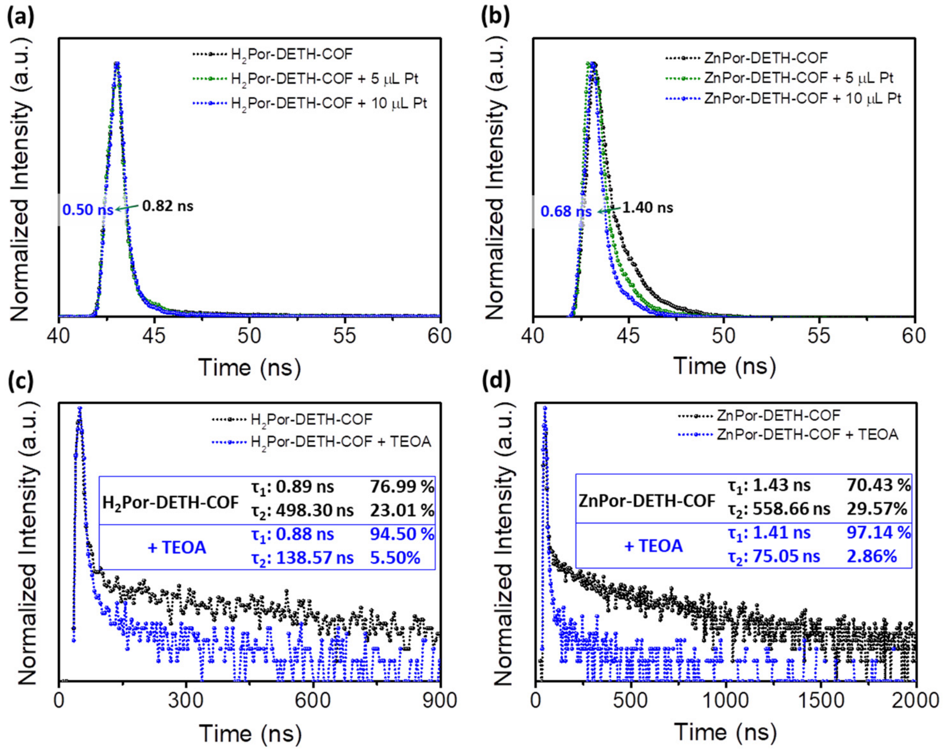

Supplementary Fig. 26 | The emission decay of H_2Por-DETH-COF and ZnPor-DETH-COF before and after adding H_2PtCl_6 and TEOA solution: (a) H_2Por-DETH-COF (2.5 mg) and (b) ZnPor-DETH-COF (2.5 mg) with addition of different volume H_2PtCl_6 (8 *wt*% solution); (c) H_2Por-DETH-COF (2.5 mg) and (d) ZnPor-DETH-COF (2.5 mg) with addition of 100 μL TEOA (1% aqueous solution).

For H_2Por-DETH-COF, after adding 10 uL H_2PtCl_6, the lifetime is shortened from 0.82 ns to 0.50 ns for H_2Por-DETH-COF and the lifetime is changed from 1.40 ns to 0.68 ns for ZnPor-DETH-COF. Since the rate of hole transfer is about two to four orders of magnitude slower than that of electrons,[4,5] the time scale between short-lived fluorescence decay and the hole migration process of COF might be mismatched, we cannot observe the quenching effect of TEOA on the COF excited state on a short lifetime scale (fluorescence decay). Taking into account the TADF process of H_2Por-DETH-COF and ZnPor-DETH-COF (*vide supra*, Supplementary Fig. 25), we observed the quenching effect of TEOA on ZnPor-DETH-COF and H_2Por-DETH-COF on a long-life time scale (vacuum condition). After adding 100 μL 1% TEOA aqueous solution, the TADF lifetimes of H_2Por-DETH-COF and ZnPor-DETH-COF decreased from

498.30 and 558.66 ns to 138.57 and 75.05 ns, respectively. Therefore, it can be concluded that the charge separation ability of ZnPor-DETH-COF is significantly better than that of H_2Por-DETH-COF.

Section 6. Photocatalysis

The H_2 photogeneration test was performed with a 20 mL pyrex tube holding MPor-DETH-COF (2.5 mg), 5 mL phosphate buffer solution (0.1 M, pH = 7.0). The suspension was ultrasonicated for 30 min before adding 2.5 μL 8 wt% H_2PtCl_6 and 50 μL triethanolamine (TEOA), and then degassing by Ar bubbling for 30 min. 600 μL CH_4 was injected into the system and functioned as the internal standard for quantitative analysis. Xe lamps (300 W) as light source for testing H_2 evolution performance, and using air fan to keep room temperature of the sample. The generated H_2 gas in the headspace of reactor was taken with a gas-tight syringe and measured by using a gas chromatograph (Shimadzu GC2014CAFC/APC) equipped with a thermal conductivity detector and a 5 Å molecular sieves GC column. Ar was used as a carrier gas.

For long term test, after each circle illumination, the samples were centrifuged and washed by water and ethanol for 3 times. And then refilled 5 mL phosphate buffer solution (0.1 M, pH = 7.0), and 50 μL TEOA, degassing by Ar bubbling for 30 min, 600 μL CH_4 was re-injected into the system for the next circle illumination.

For apparent quantum efficiency (AQE) test, 0.5 mg ZnPor-DETH-COF was dispensed in 2.5 mL PBS solution by ultrasonic, which was further added with 0.5 μL 8wt% H_2PtCl_6 (3.8wt% Pt *vs* COF) and 10 uL TEOA. After being degassed by Ar bubbling for 30 min, 300 μL CH_4 was re-injected into the system and functioned as the internal standard for quantitative analysis. By taking sodium ascorbate as sacrificial reagent, 5 mg sodium ascorbate, 2 μL 8wt% H_2PtCl_6 (15.2wt% Pt *vs* COF) were employed in the AQE test (see details in Supplementary Fig. 29, *vide infra*).

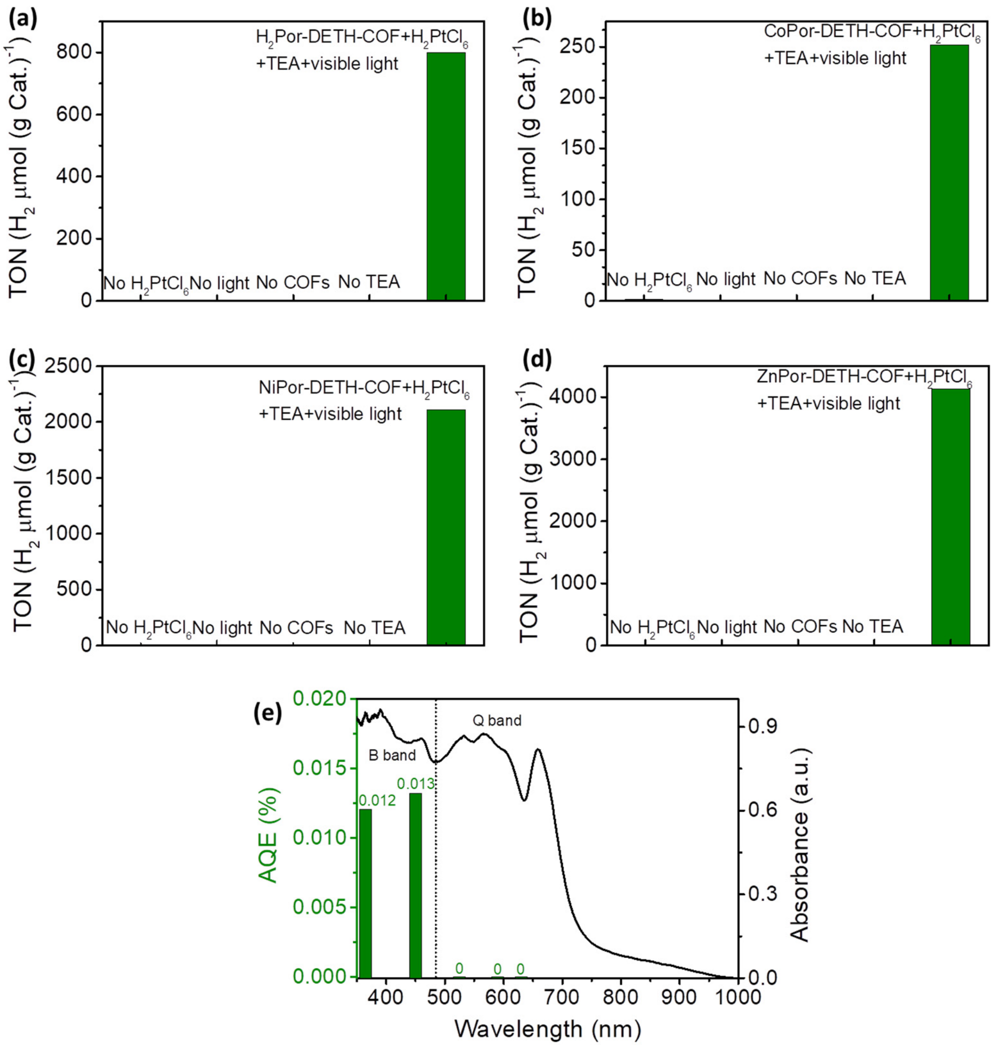

Supplementary Fig. 27 | H_2 photogeneration control experiments.

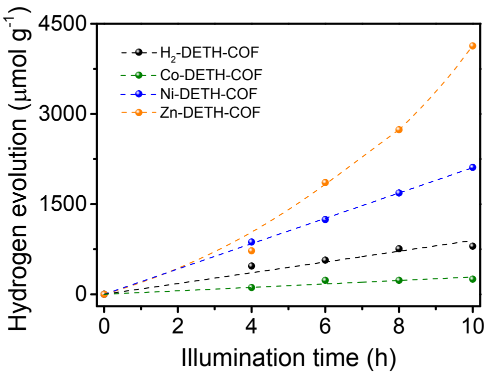

Supplementary Fig. 28 | Time dependent H_2 photogeneration using visible light for H_2Por-DETH-COF, CoPor-DETH-COF, NiPor-DETH-COF and ZnPor-DETH-COF [2.5 mg catalyst in 5 mL phosphate buffer solution, 2.5 μL (8 wt% H_2PtCl_6), 50 μL TEOA, λ > 400 nm 300 W Xe lamp].

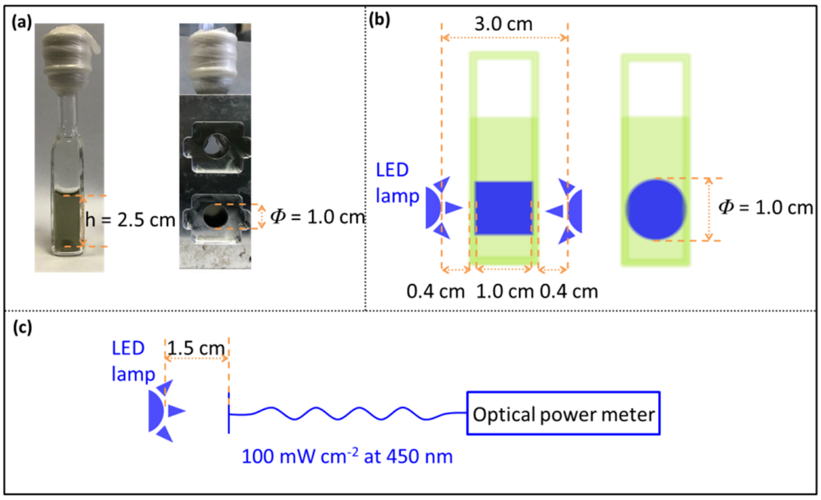

Supplementary Fig. 29 | The device for quantum efficiency measurement: (a) physical photos; (b) detailed schematic; (c) the optical power measurement process of LED lights.

With the aid of the device in Fig. 29a, we measured the quantum efficiency of ZnPor-DETH-COF, and the apparent quantum efficiency (AQE) is 0.063%. The detailed measurement process is as follows: A certain mass (0.50 mg) of ZnPor-DETH-COF, 2.5 mL PBS solution, 0.5 μL 8 wt% H_2PtCl_6 and 10 μL TEOA were sealed in the 4 mL cuvette (Fig. 29a, left) and placed in the photoreactor (Fig. 29a, right). Under the illumination of LED lamps (Fig. 29b), the H_2 amount was analyzed from gas chromatography (0.333 μmol after 30 min illumination).

$$AQE = \frac{2 \times n(H_2) \times N_A}{N_{(Incident\ Photon)}} \times 100\% \qquad (S1)$$

where $n(H_2)$ (mol) is the amount of H_2 generated in the unit time of irradiation, N_A is avogadro's constant (6.02×10^{23} mol^{-1}). $N_{(Incident\ Photon)}$ represented the number of incident photons in the unit time. The light intensity is 100 mW cm^{-2} (Fig. 29c) and the light area is 1.571 cm^2 [$\pi(\Phi/2)^2 \times 2$ side]. The N(incident Photon) could be calculated as 6.405×10^{20} (30 min) and AQE was determined as 0.063%. By taking sodium ascorbate as sacrificial reagent [reaction condition: 0.5 mg sample, 2.5 mL PBS (0.1 M, pH 7), 5 mg sodium ascorbate, 15.2 wt% Pt], the H_2 amount was determined as 1.723 μmol after 30 min illumination, and AQE was calculated as 0.32%.

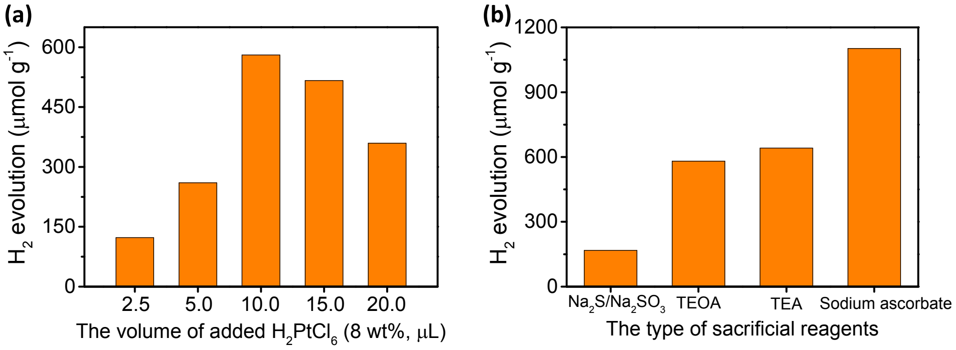

Supplementary Fig. 30 | Optimized experiment of photocatalytic H_2 generation of ZnPor-DETH-COF: (a) H_2 production versus different volume of H_2PtCl_6 (8 wt%, μL); (b) H_2 production *versus* different type of sacrificial reagents (Na_2SO_3: 0.126 mmol, Na_2S: 0.126 mmol; TEOA: 50 μL; TEA: 50 μL; Ascorbic acid: 0.126 mmol (pH~8.0); reaction time: 2 h; light source: 450 nm LED lamps).

The photocatalytic H_2 generation of ZnPor-DETH-COF in 2 hours enhanced with increasing concentration of H_2PtCl_6 (8 *wt*% aqueous solution), and reached a maximum when 10 μL H_2PtCl_6 was added (Supplementary Fig. 30a). In addition, different sacrificial reagents exhibited different hydrogen evolution activity under the same condition (Supplementary Fig. 30b).

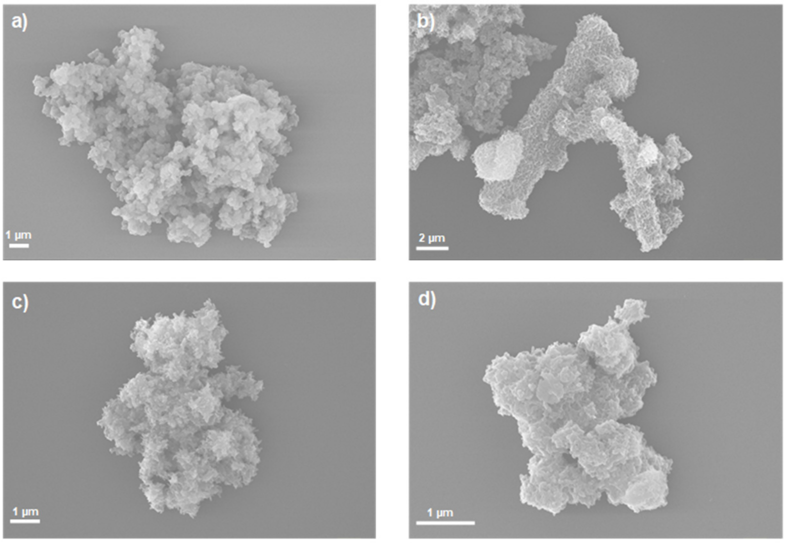

Supplementary Fig. 31 | SEM images of a) H_2Por-DETH-COF, b) CoPor-DETH-COF, c) NiPor-DETH-COF, and d) ZnPor-DETH-COF after 10 h photocatalysis experiment.

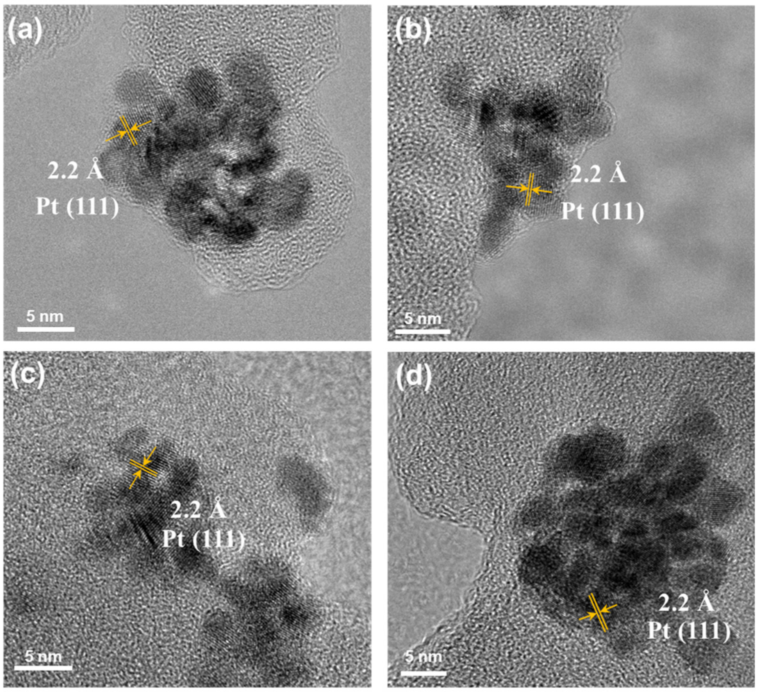

Supplementary Fig. 32 | TEM images of a) H_2Por-DETH-COF, b) CoPor-DETH-COF, c) NiPor-DETH-COF, and d) ZnPor-DETH-COF after 10 h photocatalysis experiment.

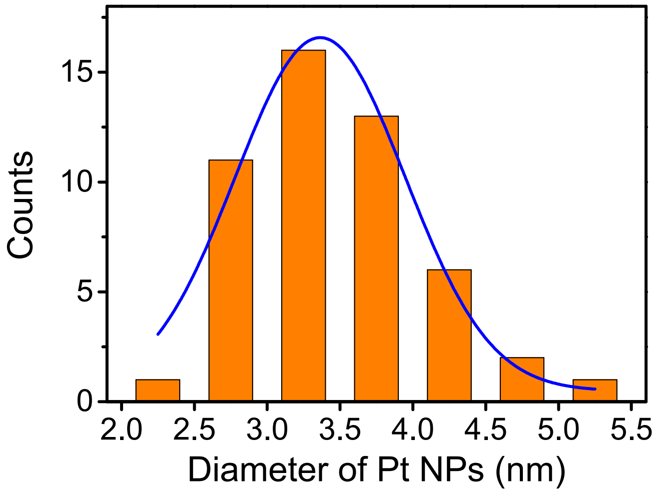

Supplementary Fig. 33 | Diameter distribution of Pt nanoparticles loaded on MPor-DETH-COF (M= H_2, Co, Ni, and Zn; statistic: 50).

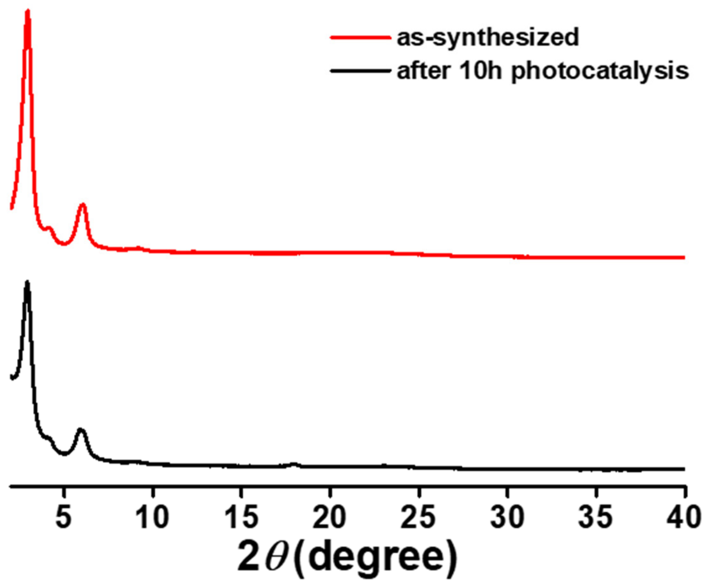

Supplementary Fig. 34 | PXRD patterns of as-synthesized H_2Por-DETH-COF (red) and recycled after 10h of photocatalysis experiment (black).

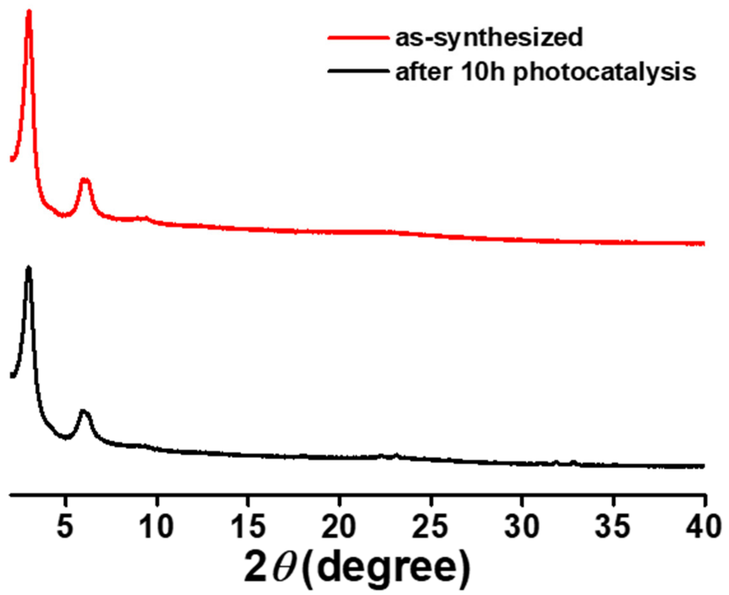

Supplementary Fig. 35 | PXRD patterns of as-synthesized CoPor-DETH-COF (red) and recycled after 10 h of photocatalysis experiment (black).

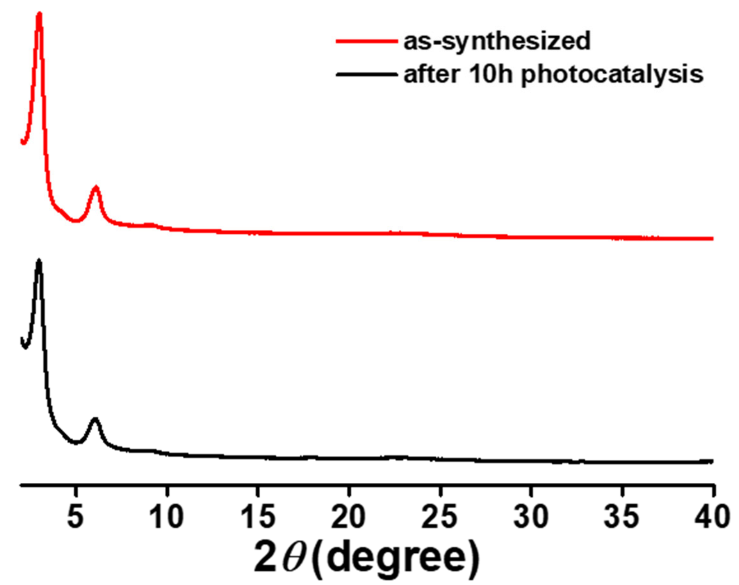

Supplementary Fig. 36 | PXRD patterns of as-synthesized NiPor-DETH-COF (red) and recycled after 10h of photocatalysis experiment (black).

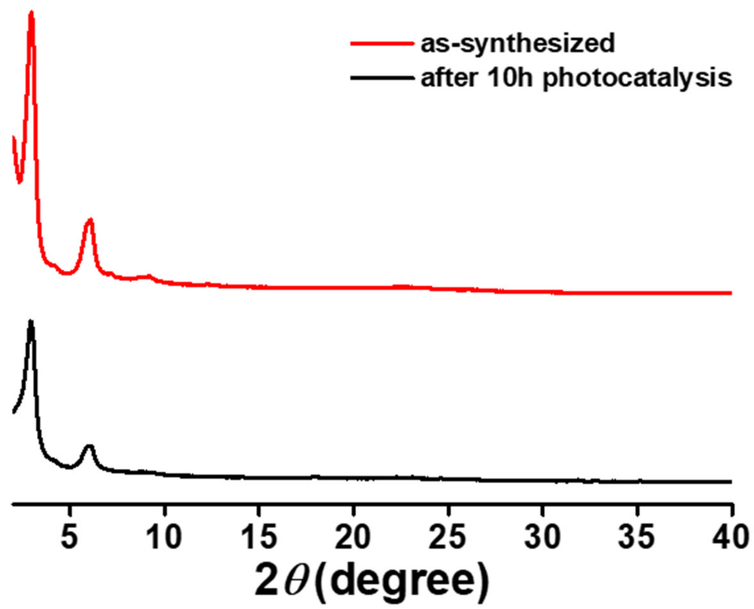

Supplementary Fig. 37 | PXRD patterns of as-synthesized ZnPor-DETH-COF (red) and recycled after 10h of photocatalysis experiment (black).

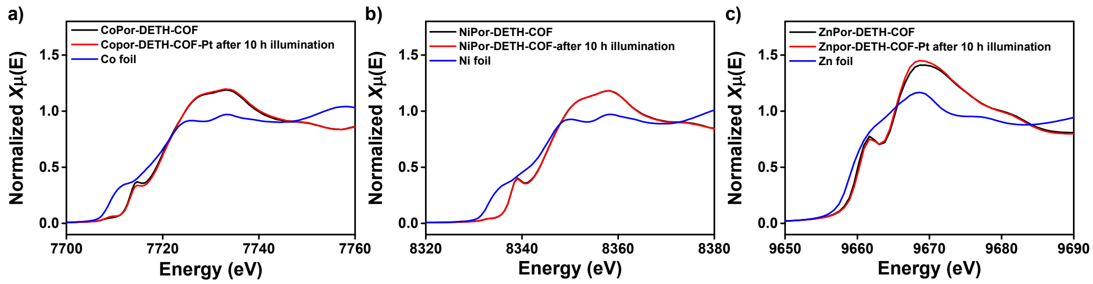

Supplementary Fig. 38 | Comparisons of XAS E-space curves before and after 10h illumination with Pt as co-catalyst: a) CoPor-DETH-COF and standard Co foil, b) NiPor-DETH-COF and standard Ni foil, c) ZnPor-DETH-COF and standard Zn foil.

Supplementary Table 7 | Fitting parameters of XAS Fourier transform R-space curves of CoPor-DETH-COF, NiPor-DETH-COF and ZnPor-DETH-COF.

**Sample**	**Atomic scatter**	**Coordination Number**	**Coordination distance (Å)**	**Debye-Waller factor (10^{-3}×Å^2)**	**ΔE_0 (eV)**
**CoPor-DETH-COF**	Co-N	4	1.96	0.32	9.65
	Co-C	8	3.00	1.66	9.65
**CoPor-DETH-COF after reaction**	Co-N	4	1.95	0.16	8.80
	Co-C	8	2.99	2.08	8.80
**NiPor-DETH-COF**	Ni-N	4	1.90	1.19	-3.00
	Ni-C	8	2.92	1.99	-3.00
**NiPor-DETH-COF after reaction**	Ni-N	4	1.94	0.73	7.45
	Ni-C	8	2.98	2.45	7.45
**ZnPor-DETH-COF**	Zn-N	4	2.05	2.56	8.44
	Zn-C	8	3.06	4.31	8.44
**ZnPor-DETH-COF after reaction**	Zn-N	4	2.06	2.59	11.09
	Zn-C	8	3.08	2.19	11.09

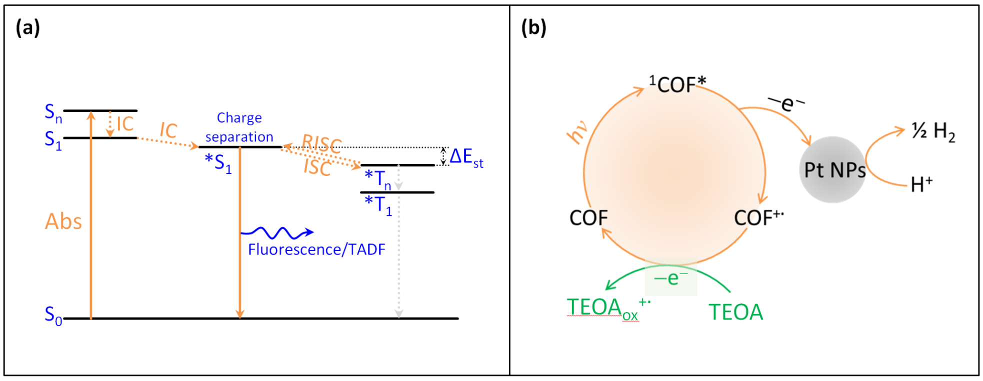

Supplementary Fig. 39 | The *Jablonski* process (a) and charge separation diagram (b) of MPor-DETH-COFs.

We didn't observe an obvious phosphorescent signal from MPor-DETH-COFs (M = H_2 and Zn) under the vacuum condition. According to the perturbation theory and compared with the monomer, the energy gap (ΔE_{st}) between the lowest singlet and triplet excited state of the polymer will be greatly reduced,[6-8] which is more conducive to the transition from $*T_n$ to $*S_1$ and returns to S_0 state to generate thermally activated delayed fluorescence (TADF). Consequently, MPor-DETH-COFs (M = H_2 and Zn) didn't show the phosphorescence and the rapid reverse intersystem crossing (RISC) process makes the charge separation process mainly occur in the excited singlet state $*S_1$.

In fact, as shown in Supplementary Fig. 25 (*vide supra*), the long-lived decay of free-base and zinc-porphyrin based COFs can be detected under the vacuum condition, which can be attributed to TADF process.[9] The weights of TADF for free-base and zinc-porphyrin based COFs are calculated as 23.0% and 29.6%. Considering the rate of hole transfer is about two to four orders of magnitude slower than that of electrons,[4,5] we infer that before ISC process, the excited state COF preferentially undergoes an oxidative quenching process with Pt NPs to produce $COF^{+•}$.[10] This fluorescence process (short-lived singlet) is an important indicator of the photocatalytic hydrogen evolution process. Then, $COF^{+•}$ is reductively quenched by acquiring an electron from TEOA to return to the ground state (Supplementary Fig. 39b).

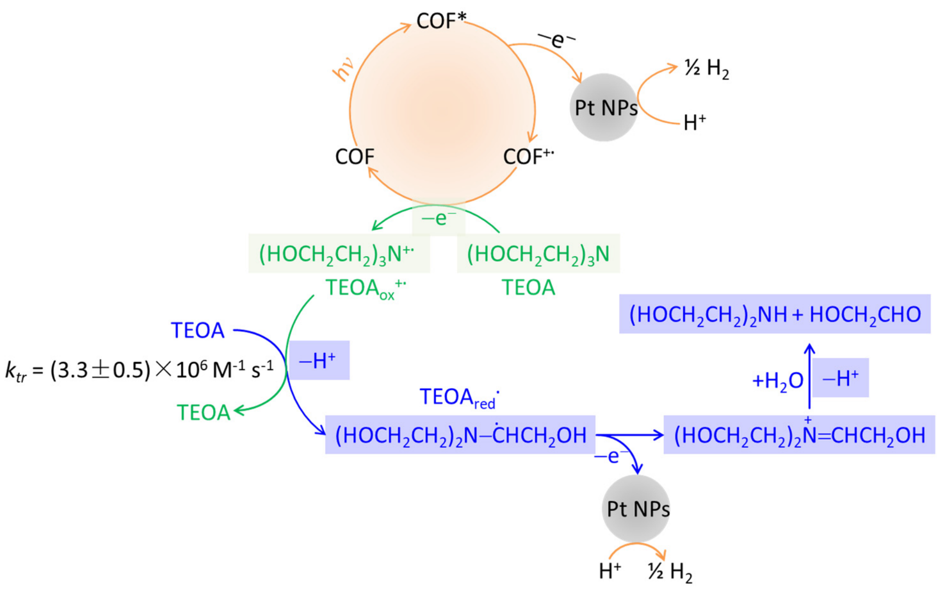

Supplementary Fig. 40 | Schematic illustration of TEOA oxidation processes.

As illustrated in Supplementary Fig. 40, the TEOA could generate two electrons and two protons. After MPor-DETH-COFs absorbed one photon, they reached to an excited state, leading to charges separation. Since the rate of hole transfer is about two to four orders of magnitude slower than that of electrons,[4,5] the excited state COF will give priority to oxidation quenching that the electrons will be transferred from COF to Pt and produce $COF^{+\bullet}$. By acquiring an electron from TEOA, COF returns to the ground state, thus completing the catalytic cycle process. Subsequently, TEOA that has lost electrons ($TEOA_{ox}^{+\bullet}$) would extract H^+ from adjacent TEOA and produce $TEOA_{red}^{\bullet}$.[11] According to the literatures,[12-15] we speculated that, in our system, $TEOA_{red}^{\bullet}$ may spontaneously lose one electron to Pt NPs and form $(HOCH_2CH_2)_2N^+{=}CHCH_2OH$ itself, which is eventually degraded into $(HOCH_2CH_2)_2NH$ and $HOCH_2CHO$ by reacting with H_2O. As a result, the above processes can be written as: $(HOCH_2CH_2)_3N + H_2O \rightarrow (HOCH_2CH_2)_2NH + HOCH_2CHO + 2H^+ + 2e^-$, under the condition that COF just gain one photon.

Section 7. DFT calculations

Adsorption energy calculations of H_2O on metalloporphyrin-based COFs. The DFT calculations were performed in the Vienna ab initio Simulation Package (VASP)[16] using the projector augmented wave (PAW)[17] method with the Perdew–Burke–Ernzerhof (PBE) exchange–correlation functional[18]. A nonlocal optB86b-vdW exchange-correlation functional[19,20] was used to describe the dispersion interaction approximately. All of the calculations were carried out as spin-polarized. The plane-wave basis kinetic energy cut-off was set to 400 eV. The porphyrin and metal atoms are allowed to be relaxed until the forces on all of the relaxed atoms were less than 0.05 eV/Å.

The adsorption energies of a water molecule (ΔE_{ad}) on metalloporphyrin-based COFs were calculated by the following equation:

$$\Delta E_{ad} = E_{H_2O\text{-}COF} - E_{COF} - E_{H_2O}$$

where $E_{H_2O\text{-}COF}$ and E_{COF} are the energies of metalloporphyrin-based COFs catalyst adsorbing a water molecule and without a water molecule adsorbed, respectively. The adsorption sites and energies of H_2O on the metalloporphyrin-based COFs are presented in Supplementary Fig. 41 and 42, respectively.

VBM, CBM and projected density of state calculations of MPor-DETH-COFs. The DFT+U calculations of Valence band maximums (VBM) and conduction band minimums (CBM) as well as projected density of state (PDOS) were performed in the Vienna ab initio Simulation Package (VASP) using the projector augmented wave (PAW) method with the Perdew–Burke–Ernzerhof (PBE) exchange–correlation functional. A nonlocal optB86b-vdW exchange-correlation functional was used to describe the dispersion interaction approximately. All of the calculations were carried out as spin-polarized. The plane-wave basis kinetic energy cut-off was set to 400 eV. The effective Coulomb (*U*) and exchange (*J*) are 2.5 eV and 1 eV. The obtained VBM and CBM of COFs are shown in Fig. 6 in the maintext and Supplementary Fig. 43 and 44. The PDOS of MPor-DETH-COFs are shown in Supplementary Fig. 45.

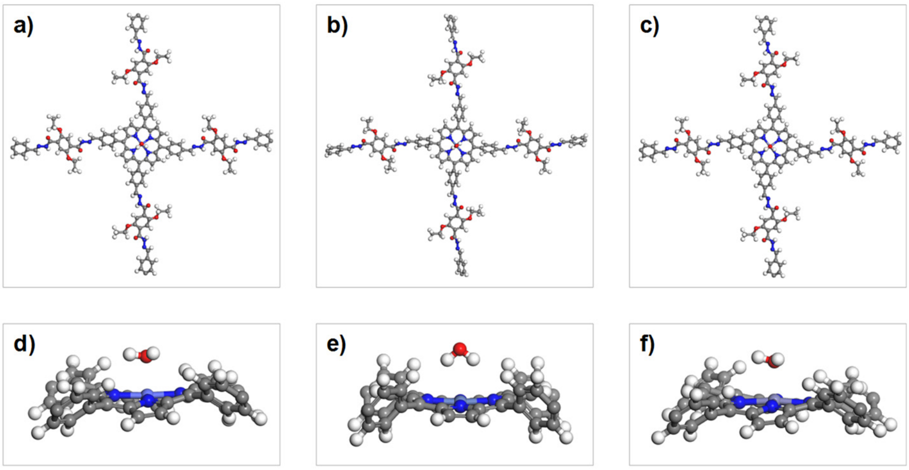

Supplementary Fig. 41 | Adsorption sites of H_2O on CoPor-DETH-COF, NiPor-DETH-COF and ZnPor-DETH-COF, respectively: a)-c) top view; d)-f) side view. The balls in different colors represent different atoms: H, white; C, grey; N, blue; O, red.

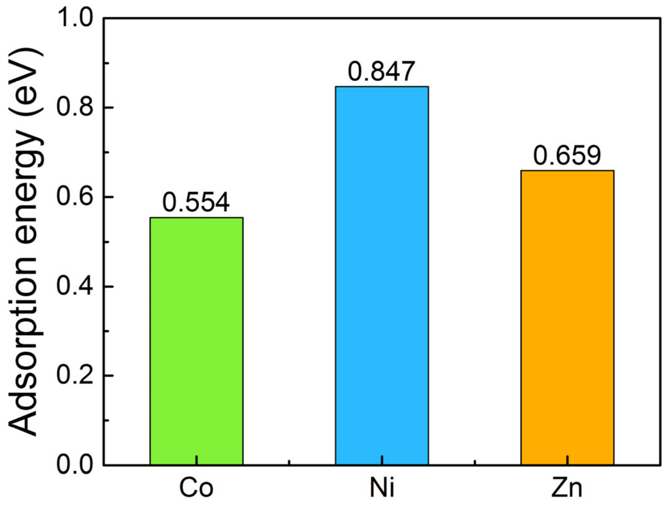

Supplementary Fig. 42 | Adsorption energies (ΔE_{ad}) of H_2O on CoPor-DETH-COF, NiPor-DETH-COF and ZnPor-DETH-COF.

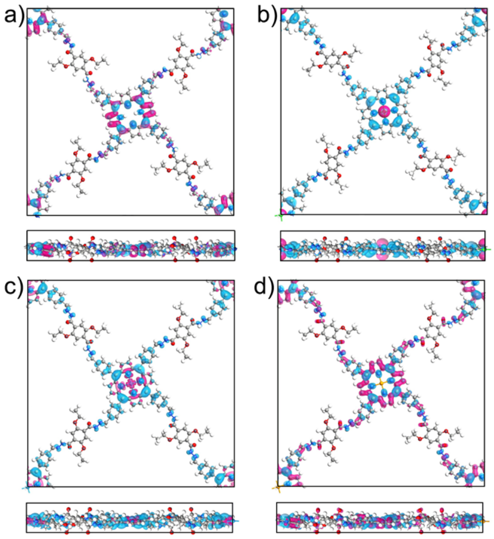

Supplementary Fig. 43 | Valence band maximums (VBM, blue) and conduction band minimums (CBM, magenta) of monolayer COFs. (a) H_2Por-DETH-COF, (b) CoPor-DETH-COF, (c) NiPor-DETH-COF and (d) ZnPor-DETH-COF. The balls in different colors represent different atoms: H, white; C, grey; N, blue; Co, olive green; Ni, light blue; Zn, orange.

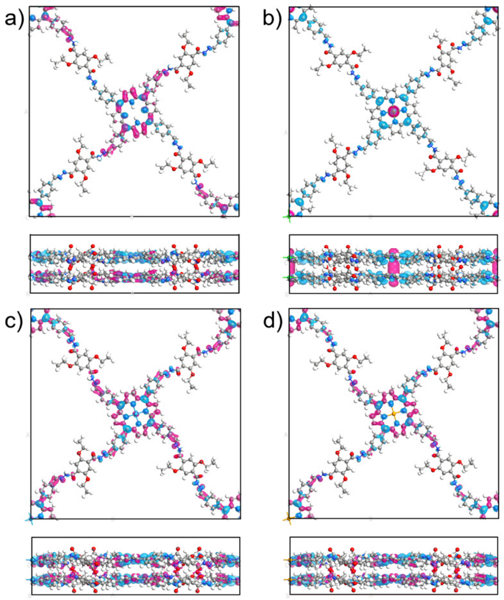

Supplementary Fig. 44 | Valence band maximums (VBM, blue) and conduction band minimums (CBM, magenta) of bilayer COFs. (a) H_2Por-DETH-COF, (b) CoPor-DETH-COF, (c) NiPor-DETH-COF and (d) ZnPor-DETH-COF. The balls in different colors represent different atoms: H, white; C, grey; N, blue; Co, olive green; Ni, light blue; Zn, orange.

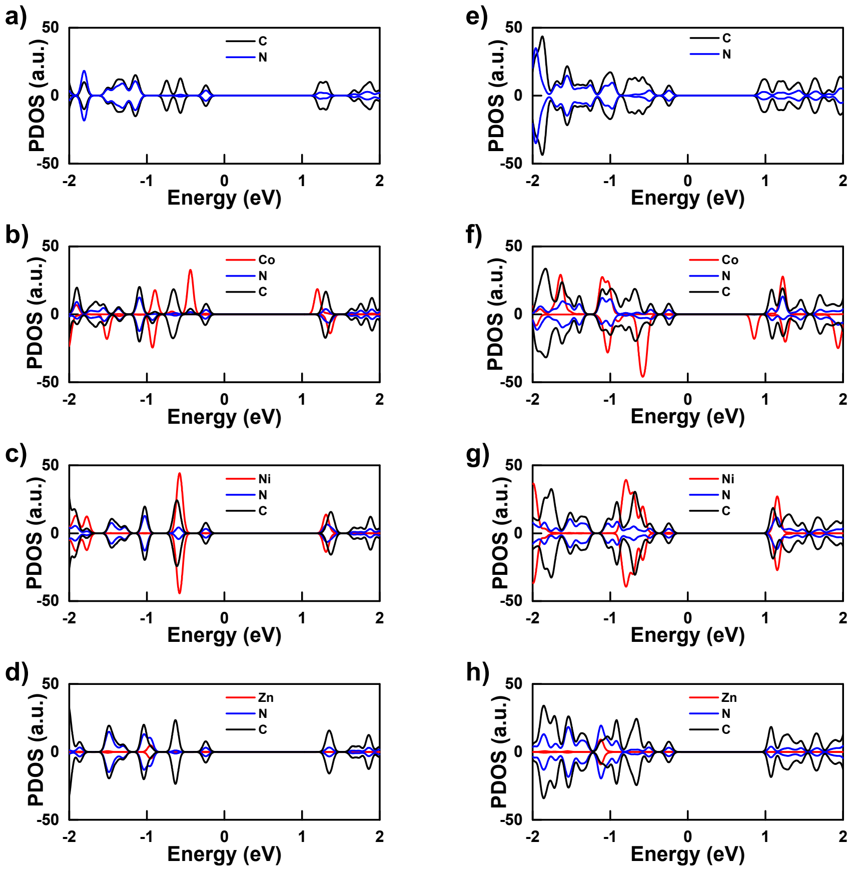

Supplementary Fig. 45 | Projected density of states (PDOS) of monolayer structures: a) H_2Por-DETH-COF, b) CoPor-DETH-COF, c) NiPor-DETH-COF and d) ZnPor-DETH-COF; AA stacked bilayer structures: e) H_2Por-DETH-COF, f) CoPor-DETH-COF, g) NiPor-DETH-COF and h) ZnPor-DETH-COF.

Supplementary Table 8 | Calculated band gaps in eV of mono- and bilayer structures from PDOS (Supplementary Fig. 45)

	**H_2Por-DETH-COF**	**CoPor-DETH-COF**	**NiPor-DETH-COF**	**ZnPor-DETH-COF**
**Monolayer**	1.10	1.06	1.18	1.20
**Bilayer**	0.83	0.73	0.96	0.94

Section 8. ^{1}H NMR Spectrum

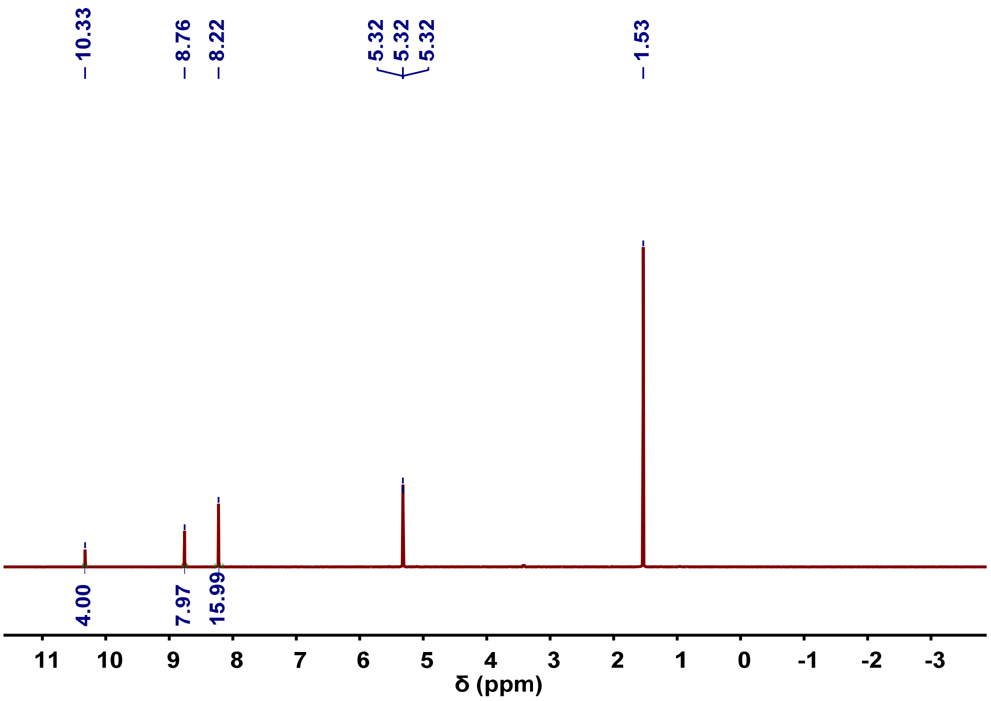

Supplementary Fig. 46 | ^{1}H NMR spectrum (CD_2Cl_2, 400 MHz) of NiPor-CHO.

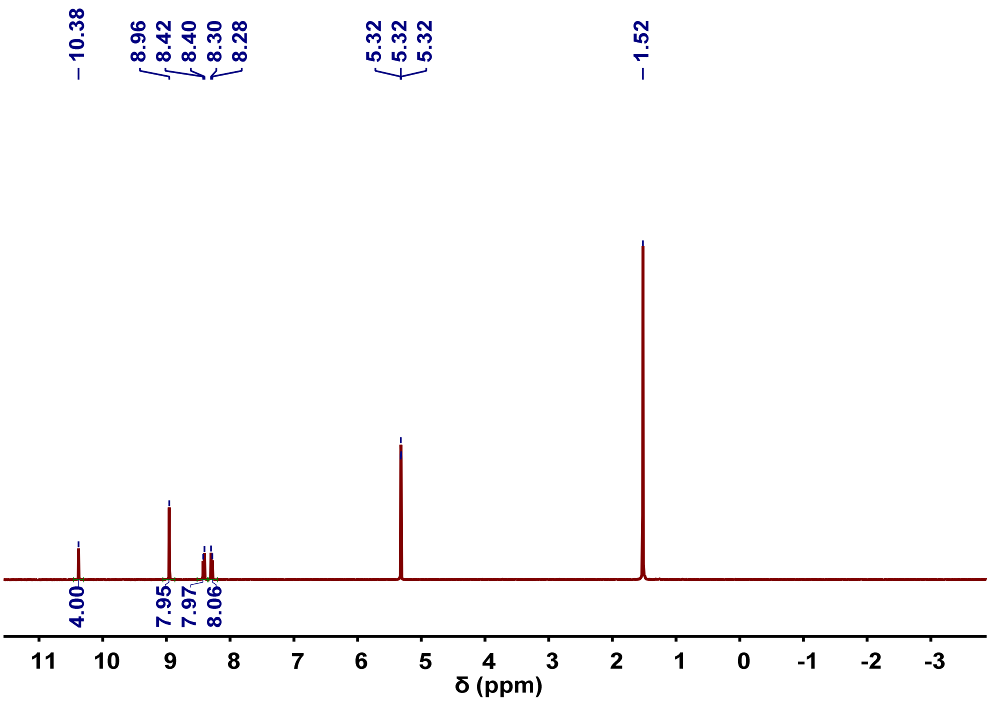

Supplementary Fig. 47 | ^{1}H NMR spectrum (CD_2Cl_2, 400 MHz) of ZnPor-CHO.

Section 9. References

1 Uribe-Romo, F. J., Doonan, C. J., Furukawa, H., Oisaki, K. & Yaghi, O. M. Crystalline covalent organic frameworks with hydrazone linkages. *J. Am. Chem. Soc.* **133**, 11478−11481 (2011).

2 Atsuhiro, O., Ben-li, L. & Kazuhiro, M. An efficient one-pot synthetic procedure of multiple porphyrin-cyclization. *Chem. Lett.* **22**, 949−952 (1993).

3 O'Keeffe, M., Peskov, M. A., Ramsden, S. J. & Yaghi, O. M. The Reticular Chemistry Structure Resource (RCSR) database of, and symbols for, crystal nets. *Acc. Chem. Res.* **41**, 1782−1789 (2008).

4 Tvrdy, K.; Frantsuzov, P. A.; Kamat, P. V. Photoinduced electron transfer from semiconductor quantum dots to metal oxide nanoparticles. *Proc. Natl. Acad. Sci. U. S. A.*, **108**, 29−34 (2011).

5 Malicki, M., Knowles, K. E. & Weiss, E. A. Gating of hole transfer from photoexcited PbS quantum dots to aminoferrocene by the ligand shell of the dots. *Chem. Commun.* **49**, 4400–4402 (2013).

6 Wang, X.-F. *et al.* Pure Organic Room Temperature Phosphorescence from Excited Dimers in Self-Assembled Nanoparticles under Visible and Near-Infrared Irradiation in Water. *J. Am. Chem. Soc.* **141**, 5045–5050 (2019).

7 Lower, S. K. & El-Sayed, M. A. The Triplet State and Molecular Electronic Processes in Organic Molecules. *Chem. Rev.* **66**, 199–241 (1966).

8 Li, J.-A. et al. Transient and Persistent Room-Temperature Mechanoluminescence from a White-Light-Emitting AIEgen with Tricolor Emission Switching Triggered by Light. *Angew. Chem. Int. Ed.* **57**, 6449–6453 (2018).

9 Tritton, D. N. et al. Iridium motif linked porphyrins for efficient light-driven hydrogen evolution via triplet state stabilization of porphyrin. *J. Mater. Chem. A* **8**, 3005–3010 (2020).

10 Giannoudis, E. *et al.* Photosensitizers for H_2 Evolution Based on Charged or Neutral Zn and Sn Porphyrins. *Inorg. Chem.* **59**, 1611−1621 (2020).

11 Georgopoulos, M. & Hoffman, M. Z. Cage escape yields in the quenching of tris (2,2'-bipyridine)ruthenium(II) by methylviologen: presence of triethanolamine as a sacrificial electron donor. *J. Phys. Chem.* **95**, 7717–7721 (1991).

12 Chan, S. F., Chou, M., Creutz, C., Matsubara, T. & Sutin, N. Mechanism of the formation of dihydrogen from the photoinduced reactions of poly(pyridine)ruthenium(II) and poly(pyridine)rhodium(III) complexes. *J. Am. Chem. Soc.* **103**, 369–379 (1981).

13 Zhu, H., Song, N., Lv, H., Hill, C. L. & Lian, T. Near Unity Quantum Yield of Light-Driven Redox Mediator Reduction and Efficient H_2 Generation Using Colloidal Nanorod Heterostructures. *J. Am. Chem. Soc.* **134**, 11701–11708 (2012).

14 Wu, K., Zhu, H. & Lian, T. Ultrafast Exciton Dynamics and Light-Driven H_2 Evolution in Colloidal Semiconductor Nanorods and Pt-Tipped Nanorods. *Acc. Chem. Res.* **48**, 851–859 (2015).

15 Yang, X.-J., Chen, B., Zheng, L.-Q., Wu, L.-Z. & Tung, C.-H. Highly efficient and selective photocatalytic hydrogenation of functionalized nitrobenzenes. *Green Chem.* **16**, 1082–1086 (2014).

16 Kresse, G., Furthmüller, J. Efficient iterative schemes for ab initio total-energy calculations using a plane-wave basis set. *J., Phys. Rev. B: Condens. Matter Mater. Phys.* **54**, 11169−11186 (1996).

17 Blöchl, P. E. Projector augmented-wave method. *Phys. Rev. B: Condens. Matter Mater. Phys.* **50**, 17953−17979 (1994).

18 Perdew, J. P., Burke, K., Ernzerhof, M. Generalized gradient approximation made simple. *Phys. Rev. Lett.* **77**, 3865 (1996).

19 Klimes, J., Bowler, D. R., Michaelides, A. Chemical accuracy for the van der Waals density functional. *J. Phys.: Condens. Matter*. **22**, 022201 (2010).

20 Klimes, J., Bowler D. R., Michaelides, Van der Waals density functionals applied to solids. *A. Phys. Rev. B: Condens. Matter Mater. Phys.*, **83**, 195131 (2011).
